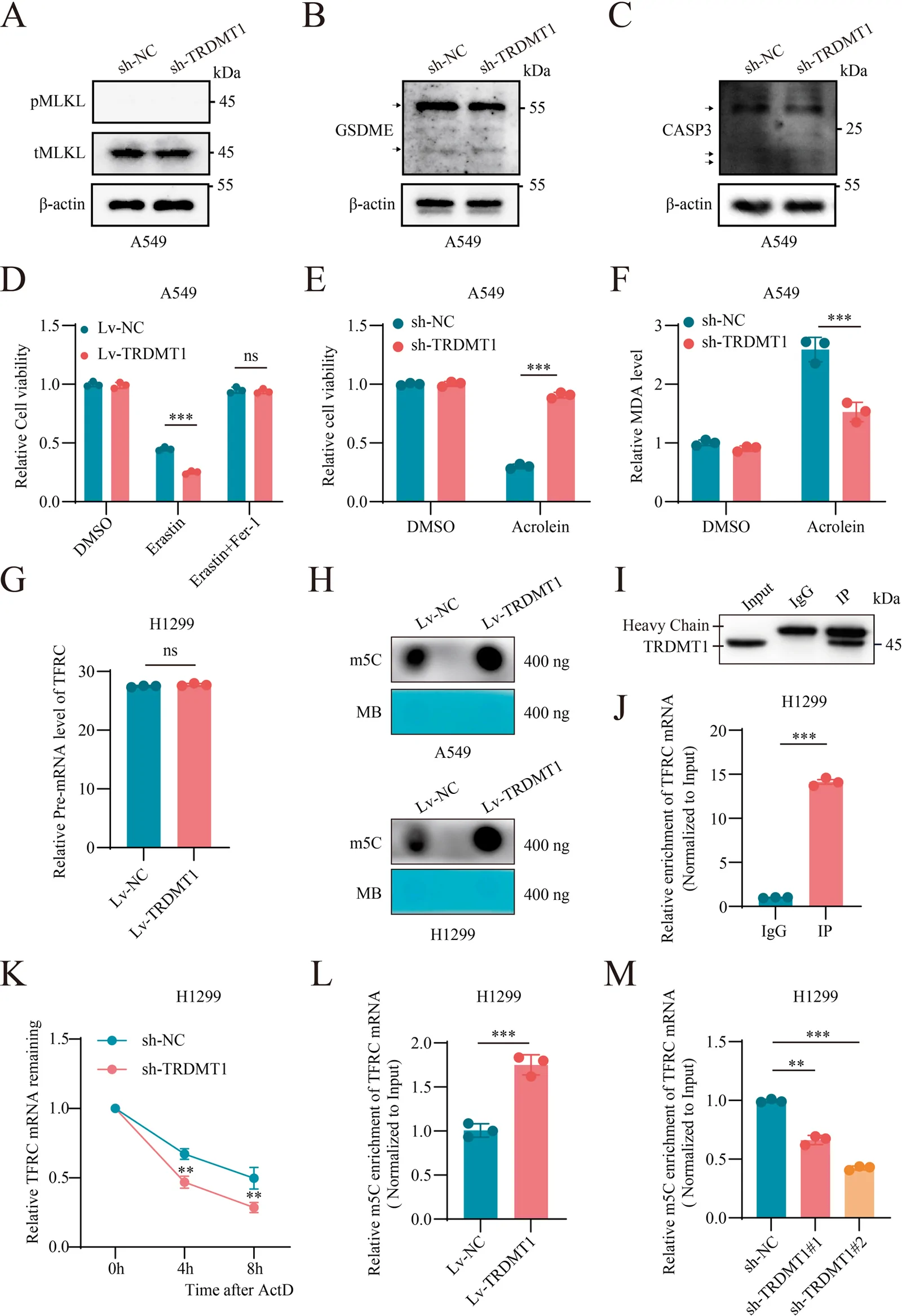# TRDMT1 slows lung cancer growth by stabilizing *TFRC* mRNA and triggering ferroptosis cell death

**DOI:** 10.1016/j.jbc.2026.113474

**Published:** 2026-08-24

**Authors:** Ying Zhu, Zuli Jiang, Youming Chen, Hongchun Liu, Xuanzhi Wang, Hongshen Lu, Yang Li, Xiangzhuan Zhao, Ruike Zhang, Lingxiu Meng, Keyu Jin, Zeduo Xu, Longfeng Zhang, Zheng Wu, Kanghui Wu, Hongyang Liu, Huihui Gu, Junhu Wan

**Affiliations:** 1 Department of Clinical Laboratory, The First Affiliated Hospital of Zhengzhou University, Zhengzhou, Henan, China; 2 Department of Obstetrics and Gynecology, The Third Affiliated Hospital of Zhengzhou University, Zhengzhou, Henan, China; 3 School of Basic Medical Sciences, Zhengzhou University, Zhengzhou, Henan, China; 4 Department of Blood Transfusion, Children's Hospital Affiliated to Zhengzhou University, Zhengzhou, Henan, China; 5 Zhengzhou Health College, Zhengzhou, Henan, China; 6 School of Life Science, Zhengzhou University, Zhengzhou, Henan, China

**Keywords:** TRDMT1, TFRC, lung cancer, m5C methylation, ferroptosis

## Abstract

Lung adenocarcinoma (LUAD), the most common subtype of non-small cell lung cancer, remains a significant therapeutic challenge due to its high mortality rates, driven by both inherent and acquired resistance to standard therapies. Emerging evidence highlights the role of epitranscriptomic regulation, particularly RNA modifications such as 5-methylcytosine (m5C), in the pathogenesis of cancer. This study identifies TRDMT1 (DNMT2), an m5C methyltransferase, as a tumor suppressor in LUAD. It was found that TRDMT1 expression is significantly lower in LUAD tissues, and this reduction is associated with poor prognosis in patients. Functional assays indicated that TRDMT1 inhibits the proliferation, migration, and invasion of LUAD cells *in vitro*. Mechanistically, transcriptomic profiling and subsequent investigation revealed that TRDMT1 enhances the stability of transferrin receptor (*TFRC*) mRNA in an m5C-dependent manner. This post-transcriptional regulation leads to TFRC upregulation, which subsequently disrupts intracellular iron homeostasis, culminating in increased susceptibility to ferroptosis—an iron-dependent form of regulated cell death. Rescue experiments confirmed that the tumor-suppressive and pro-ferroptotic effects of TRDMT1 are mediated through TFRC. This study unveils a novel TRDMT1-TFRC regulatory axis that suppresses LUAD progression through the modulation of ferroptosis, highlighting this pathway as a promising therapeutic target for future interventions.

Lung cancer remains the leading cause of cancer-related mortality and incidence worldwide, representing a persistent and significant global health burden (1). The disease is characterized by substantial heterogeneity in its pathological subtypes and clinical stages, which in turn dictates divergent treatment strategies and prognostic outcomes. Lung cancer is primarily divided into two major categories—small cell lung carcinoma (SCLC) and non-small cell lung carcinoma (NSCLC)—to standardize clinical management and prognostic assessment. NSCLC constitutes about 85% of all lung cancer cases (2, 3). Among NSCLC subtypes, LUAD has emerged as the most prevalent histological form, constituting up to 40% of all lung cancers (4). The high mortality rate associated with LUAD is largely attributable to its aggressive biological behavior and the frequent diagnosis at advanced, inoperable stages (5, 6). Over the past decade, the advent of targeted therapies and immune checkpoint inhibitors has improved survival outcomes for a subset of patients with LUAD, particularly those harboring actionable driver mutations or exhibiting high PD-L1 expression. Despite these considerable advances, the overall five-year survival rate for NSCLC remains unsatisfactory, and the global incidence of lung cancer continues to rise (7, 8, 9). Critical challenges in LUAD management persist, including both intrinsic and acquired therapeutic resistance, as well as high rates of relapse and metastasis (10, 11). Addressing these ongoing obstacles requires a deeper understanding of the molecular pathogenesis of LUAD, which will be essential for developing innovative strategies to overcome therapeutic resistance and prevent disease recurrence and metastasis.

RNA chemical modifications represent crucial post-transcriptional regulatory mechanisms within the epitranscriptome. Among over 170 identified modifications, N6-methyladenosine (m6A) and 5-methylcytosine (m5C) function through dynamic "writer-eraser-reader" machinery to fine-tune RNA metabolism, stability, and translation (12). The m5C modification, catalyzed by NSUN family methyltransferases and recognized by specific readers, enables precise control of RNA function across multiple RNA species (13, 14, 15). Its dysregulation is strongly implicated in carcinogenesis and other pathologies (16, 17, 18). TRDMT1 constitutes a distinctive RNA methyltransferase within the m5C machinery, characterized by its unique structural features and substrate specificity (19). Despite structural homology with DNA methyltransferases, TRDMT1 predominantly targets transfer RNA, particularly modifying the C38 site of tRNA [Asp] to maintain structural integrity and translational fidelity under stress conditions (20). Emerging evidence indicates TRDMT1's catalytic activity extends to mRNA, rRNA, and non-coding RNAs, implicating it in broader regulatory networks (21). In oncological contexts, TRDMT1 demonstrates multifaceted roles: it promotes ovarian tumorigenesis *via* *APC* mRNA methylation and β-catenin activation (22), correlates with advanced gastric cancer progression (23), and modulates the osteosarcoma microenvironment through STING pathway regulation (24). Additional studies reveal its involvement in DNA damage response and genomic stability maintenance through both catalytic and non-catalytic mechanisms. While TRDMT1 is established as a contributor to cancer progression through RNA stability regulation, DNA damage response, and immune modulation, its biological functions in lung cancer pathogenesis remain largely unexplored, particularly regarding potential cross-talk with ferroptosis and iron metabolic pathways.

Ferroptosis is an iron-dependent form of regulated cell death driven by the lethal accumulation of lipid peroxides following the collapse of antioxidant defenses (25). This process is initiated through extrinsic or intrinsic pathways and governed by an integrated network of iron, lipid, and amino acid metabolism (26, 27). Central to its mechanism is ferrous iron (Fe^2+^), which catalyzes lipid peroxidation *via* the Fenton reaction, targeting polyunsaturated fatty acid-containing phospholipids. The system Xc^-^–GSH–GPX4 axis constitutes the primary cellular defense, wherein glutathione depletion and iron overload synergistically promote ferroptotic cell death (28). Pharmacological agents such as Erastin and RSL3 exemplify targeted induction of ferroptosis and serve as probes for mechanistic dissection (29, 30). In lung cancer, ferroptosis has emerged as a regulatory mechanism with therapeutic implications for overcoming treatment resistance (31, 32). For instance, USP35 stabilizes ferroportin to enhance iron export and suppress ferroptosis (33), while the m5C methyltransferase NSUN2 promotes ferroptosis sensitivity by stabilizing NRF2 (34). Similarly, YTHDC2 inhibits the system Xc^−^ subunit SLC3A2 *via* HOXA13 downregulation, promoting ferroptosis and suppressing tumor progression (35). These findings illuminate a sophisticated regulatory circuitry, positioning ferroptosis as a promising target for lung cancer intervention. Nonetheless, the role of TRDMT1 in this process remains unexplored and merits further investigation.

In this study, we demonstrated that TRDMT1 is significantly downregulated in lung adenocarcinoma tissues, and its low expression is closely associated with poor patient prognosis. *In vitro* and *in vivo* functional experiments revealed that TRDMT1 effectively suppresses the proliferation, migration, and invasion of lung cancer cells. Mechanistically, we found that TRDMT1 enhances the stability of *TFRC* mRNA in an m5C-dependent manner, leading to upregulated TFRC expression, which subsequently increases cellular susceptibility to ferroptosis and thereby restrains tumor progression. These findings offer novel perspectives on RNA epigenetic regulation in lung cancer, highlighting the potential of TRDMT1 as a therapeutic target for lung adenocarcinoma.

## Results

### Comprehensive profiling of m5C regulators identifies a prognostic signature in lung adenocarcinoma

To evaluate the clinical significance of m5C RNA methylation in lung cancer, we systematically analyzed the expression patterns of 11 key m5C regulators (writers, erasers, and readers: *YBX1*, *ALYREF*, *NSUN5*, *NSUN2*, *NOP2*, *NSUN3*, *TET3*, *NSUN6*, *TRDMT1*, *ALKBH1*, and *NSUN4*) using data from The Cancer Genome Atlas (TCGA). As illustrated in Figure 1, *A* and *B*, several regulators exhibited significant alterations in expression. Subsequent survival analysis categorized these genes as either risk or favorable factors based on their correlation with patient outcomes (Fig. 1*C*). Univariate Cox proportional hazards regression analysis was utilized to evaluate the individual prognostic value of these regulators. Among the candidates, *TRDMT1*, *NOP2*, *NSUN4*, and *ALYREF* showed significant associations with overall survival (Fig. 1*D*). A risk score was subsequently constructed based on the expression profiles of these four genes. To evaluate the prognostic value of this risk score relative to conventional clinical parameters, we performed further univariate and multivariate Cox regression analyses. Univariate analysis revealed that clinical stage, T stage, N stage, and the risk score were significantly associated with overall survival (Fig. 1*E*). Subsequent multivariate adjustment confirmed that the risk score, alongside clinical stage, remained an independent predictor of poor survival (Fig. 1*F*). Importantly, the risk score exhibited the highest hazard ratio among all variables assessed. Based on the multivariate Cox regression results, a nomogram was constructed integrating the risk score and key clinical parameters, including clinical stage, to predict 1-, 3-, and 5-years survival probabilities (Fig. 1*G*). We next evaluated the diagnostic performance of this model using receiver–operating characteristic (ROC) curves. While clinical and N stages were the strongest individual predictors (AUC = 0.693 and 0.658, respectively; Fig. 1*H*), the risk score was comparable to T stage and superior to age, gender and M stage, but inferior to stage and N stage. Furthermore, time-dependent ROC analysis yielded AUC values of 0.641, 0.647, and 0.599 for predicting 1-, 3-, and 5-years survival, respectively (Fig. 1*I*). While these values indicate modest predictive power for standalone clinical application, the signature effectively captures the biological relevance of m5C regulators in LUAD.

**Figure 1:**
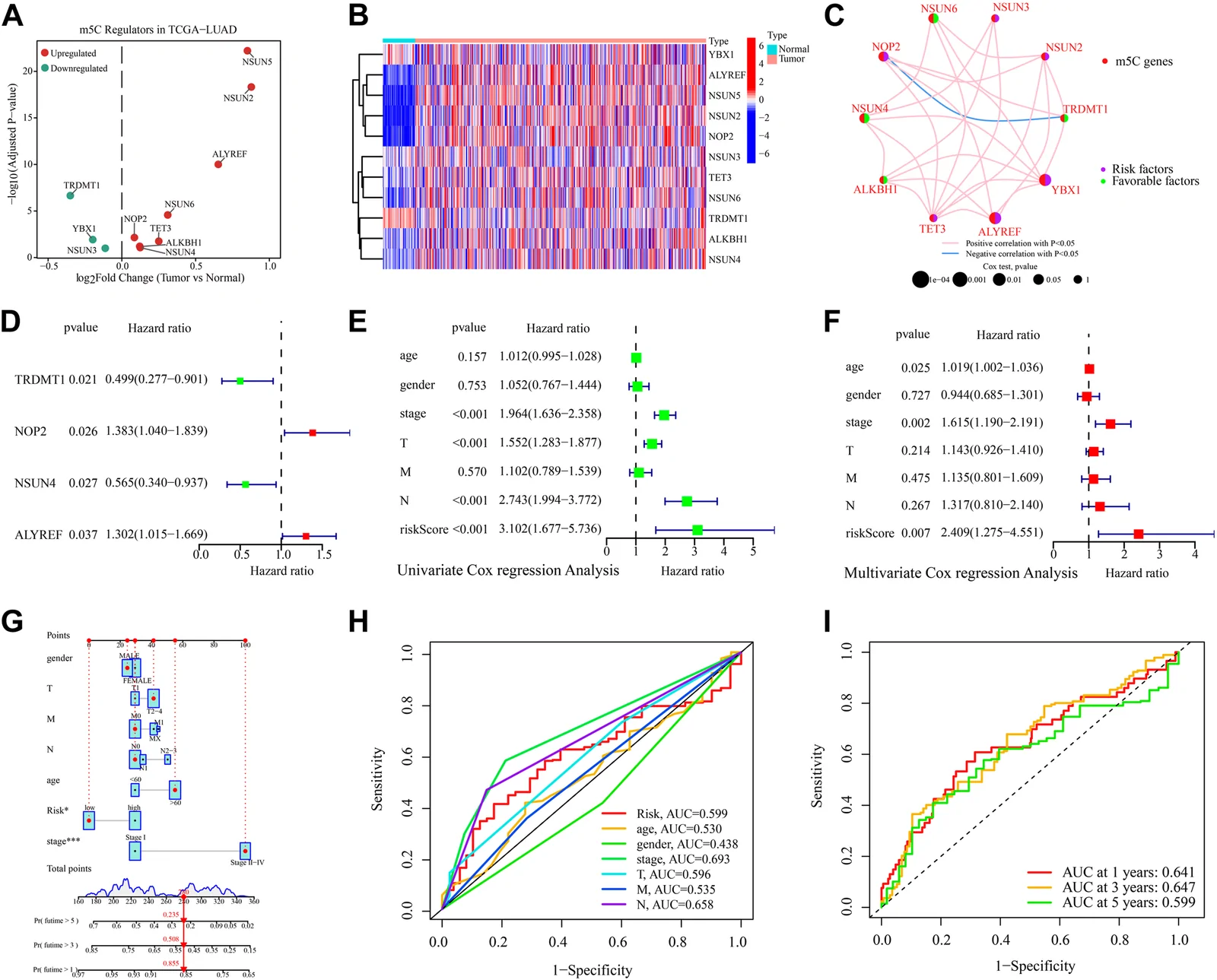
**Comprehensive profiling of m5C regulators identifies a prognostic signature in lung adenocarcinoma.***A*, expression profiling of 11 selected m5C regulators in LUAD (*n* = 483) compared to normal tissues (*n* = 59) from the TCGA-LUAD cohort. Upregulated (log2FC > 0) and downregulated (log2FC < 0) genes are highlighted in *red* and *green*, respectively. *B*, heatmap illustrating their differential expression patterns. Colors represent z-score transformed expression values. *C*, Correlation network of the 11 m5C regulators. Node size indicates prognostic significance (*p*-value from log-rank test), and lines denote positive (*red*) or negative (*blue*) correlations (*p* < 0.05). *D*, Forest plot detailing hazard ratios (HR) and 95% confidence intervals (CI) for overall survival associated with four key m5C regulators (*TRDMT1, NOP2, NSUN4, ALYREF*) derived from univariate Cox regression. *E* and *F*, Forest plots of univariate (*E*) and multivariate (*F*) analyses evaluating the independent prognostic value of the constructed risk score alongside clinical characteristics (age, gender, stage, T, M, N). *G*, a predictive nomogram integrating the risk score and clinical stage to estimate 1-, 3-, and 5-years overall survival probabilities. *H*, Receiver–operating characteristic (ROC) curves at 5 years comparing the predictive power (Area Under the Curve, AUC) of the risk score against conventional clinical parameters. *I*, time-dependent ROC curves assessing the predictive accuracy of the risk score for 1-, 3-, and 5-years survival.

### TRDMT1 is frequently downregulated and exhibits tumor-suppressive potential in LUAD

Among the RNA m5C regulatory factors analyzed, *TRDMT1* uniquely emerged as a favorable prognostic factor. Therefore, we focused on investigating its potential tumor-suppressive role in lung cancer. To systematically evaluate *TRDMT1* expression in LUAD, we analyzed transcriptomic data from the TCGA-LUAD cohort and independent GEO datasets. *TRDMT1* mRNA levels were consistently downregulated in LUAD tissues compared to normal samples, including in matched tumor–adjacent normal pairs (Fig. 2, *A* and *B*, *F–G*). Furthermore, decreased *TRDMT1* expression was associated with higher T and N stages in a subset of comparisons in the TCGA-LUAD cohort (Fig. 2, *C* and *D*). Low *TRDMT1* expression predicted poorer overall survival in the TCGA cohort (Fig. 2*E*), with a consistent but non-significant trend in GSE31210 (log-rank *p* = 0.0605; Fig. 2*H*). To experimentally validate these findings at the protein level, we performed immunohistochemical (IHC) staining on 15 paired LUAD and adjacent non-tumor tissues. TRDMT1 protein expression was lower in tumor tissues (Fig. 2*I*). Subsequent blinded semi-quantitative scoring confirmed that the immunoreactive score (IRS) of TRDMT1 was significantly downregulated in the tumor tissues compared to the normal controls (Fig. 2*J*). Western blot analysis further corroborated these results, showing consistent downregulation of TRDMT1 in LUAD tissues relative to matched normal samples (Fig. 2*K*).

**Figure 2:**
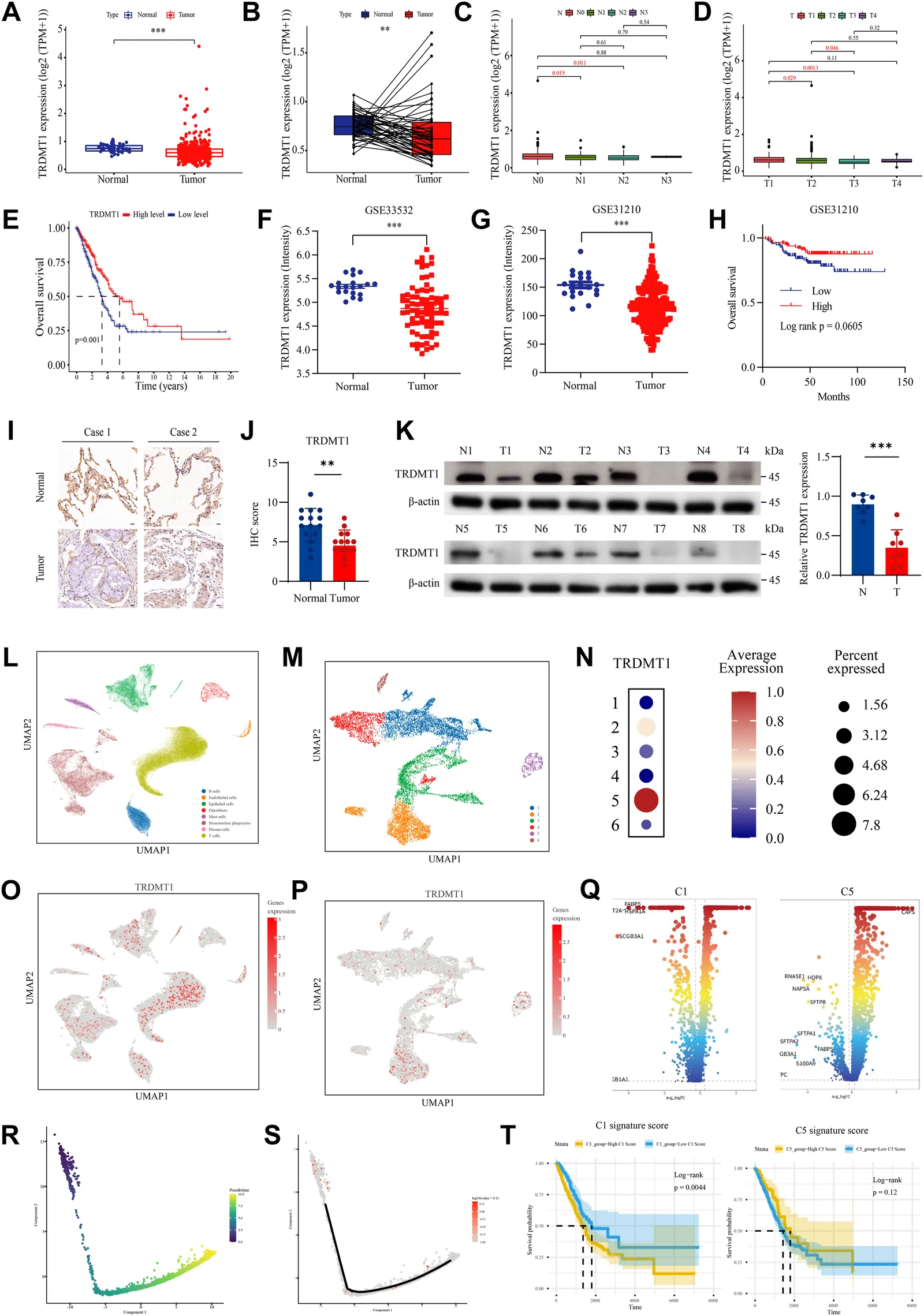
**TRDMT1 is frequently downregulated and exhibits tumor-suppressive potential in LUAD.***A* and *B*, unpaired (*A*) and paired (*B*) analyses of *TRDMT1* mRNA expression in LUAD and normal tissues from the TCGA dataset. *C* and *D*, Box plots showing the correlation between *TRDMT1* expression and clinical N-stage (*C*) and T-stage (*D*) in the TCGA-LUAD cohort. *E*, Kaplan–Meier overall survival curve of LUAD patients in the TCGA cohort, stratified by high and low *TRDMT1* expression (log-rank test). *F* and *G*, validation of *TRDMT1* downregulation in independent GEO datasets: GSE33532 (*F*) and GSE31210 (*G*). *H*, Kaplan-Meier survival analysis for the GSE31210 cohort. *I*, representative IHC staining images of TRDMT1 in human LUAD and adjacent normal tissues. Scale bar = 100 μm. *J*, statistical analysis of TRDMT1 IHC scores in paired normal and tumor tissues (n = 15 pairs). Data represent consensus IRS scores, which were reached by two blinded observers through an initial independent evaluation followed by a joint review. *K*, Western blot analysis and densitometric quantification of TRDMT1 protein levels in paired LUAD (T) and adjacent normal (N) tissue lysates from eight patients. β-actin served as a loading control. *L* and *M*, Uniform Manifold Approximation and Projection (UMAP) visualizations of 84,338 high-quality single cells from GSE189357 and GSE131907, showing global clustering (*L*) and subclustering of the epithelial cell population (*M*). *N*, Dot plot showing the expression levels and percentage of cells expressing *TRDMT1* across the epithelial subclusters in (*M*). *O* and *P*, Feature plots highlighting *TRDMT1* expression distribution in global cell clusters (*O*) and epithelial subclusters (*P*). *Q*, Volcano plots illustrating the differentially expressed genes (DEGs) in epithelial subclusters C1 and C5. *R* and *S*, Pseudotime developmental trajectory of epithelial cells reconstructed by Monocle (*R*), overlaid with the dynamic expression of *TRDMT1* (*S*). *T*, Kaplan-Meier survival analysis based on the gene expression signatures of C1 and C5. Data are presented as mean ± SD. ∗*p* < 0.05, ∗∗*p* < 0.01, ∗∗∗*p* < 0.001, determined by Student's *t* test/Wilcoxon test (*A*, *F* and *G*), paired *t* test (*B*, *J* and *K*), or one-way ANOVA/Kruskal-Wallis test (*C* and *D*).

To dissect the expression pattern of *TRDMT1* across distinct cell subpopulations at single-cell resolution, we analyzed the integrated GEO datasets GSE131907 and GSE189357. After stringent quality control, a total of 84,338 high-quality cells were retained for downstream analyses (Fig. S1, *A–D*). Dimensionality reduction and cell type annotation identified eight major cell types (Fig. 2*L*). Focusing on the epithelial compartment, we isolated 11,374 tumor epithelial cells and performed reclustering, which partitioned them into six transcriptionally distinct subgroups (Fig. 2*M*). *TRDMT1* expression exhibited notable subpopulation specificity. Bubble plots and UMAP visualizations revealed that *TRDMT1* was most highly expressed in subgroup C5, whereas its expression was lower in C1 and C4 (Fig. 2, *N–P*). To characterize the functional identities of C1 and C5, differential gene expression analysis identified their respective significantly upregulated marker genes (Fig. 2*Q*). Pseudotemporal trajectory inference further revealed a continuous spectrum of cellular states, ordered from an initial (dark blue) to a terminal state (yellow) (Fig. 2*R*). Along this trajectory, *TRDMT1* expression was elevated in earlier states but declined in later stages, suggesting its potential involvement in modulating phenotypic transitions of lung cancer cells (Fig. 2S). To assess the clinical relevance of these subpopulations, C1 and C5 signature scores were computed for each patient based on their respective differentially expressed gene sets. Kaplan–Meier analysis indicated that patients with high C1 signature scores had shorter overall survival, whereas high C5 signature scores showed no significant association with overall survival (log-rank *p* = 0.12), although the direction was consistent with a favorable effect (Fig. 2*T*). Collectively, these findings reveal substantial intra-tumoral heterogeneity of *TRDMT1* within the LUAD epithelial compartment, where progressive loss of *TRDMT1* correlates with a more advanced cellular state and poorer clinical outcomes.

### TRDMT1 suppresses oncogenic phenotypes in LUAD cells *in vitro*

To elucidate the biological functions of TRDMT1 in LUAD, we established stable *TRDMT1*-overexpressing and -knockdown A549 and H1299 cell lines. Successful ectopic expression and efficient silencing of *TRDMT1* were confirmed at both the mRNA and protein levels (Fig. S2, *A–D*). We subsequently investigated the influence of TRDMT1 on key oncogenic properties. EdU incorporation assays revealed that *TRDMT1* overexpression inhibited proliferation in both cell lines (Figs. 3*A* and S3*A*), whereas *TRDMT1* depletion significantly enhanced their proliferative capacity (Figs. 3*B* and S3*B*). Further supporting these observations, CCK-8 proliferation assays indicated a substantial reduction in cellular viability upon *TRDMT1* upregulation (Figs. 3*C* and S3*C*), in contrast to an accelerated growth rate in *TRDMT1*-deficient cells (Figs. 3*D* and S3*D*). In parallel, colony formation assays showed a significant impairment in the clonogenic potential of *TRDMT1*-overexpressing cells (Figs. 3*E* and S3*E*), while TRDMT1 silencing led to a marked elevation in colony numbers (Figs. 3*F* and S3*F*). In addition to its anti-proliferative effects, TRDMT1 was found to considerably attenuate the migratory and invasive properties of LUAD cells. Transwell migration and Matrigel-based invasion assays collectively demonstrated that *TRDMT1* overexpression led to a suppression of both migratory and invasive capacities (Figs. 3*G* and S3*G*). Conversely, *TRDMT1* knockdown substantially augmented these migratory and invasive behaviors (Figs. 3*H* and S3*H*).

**Figure 3:**
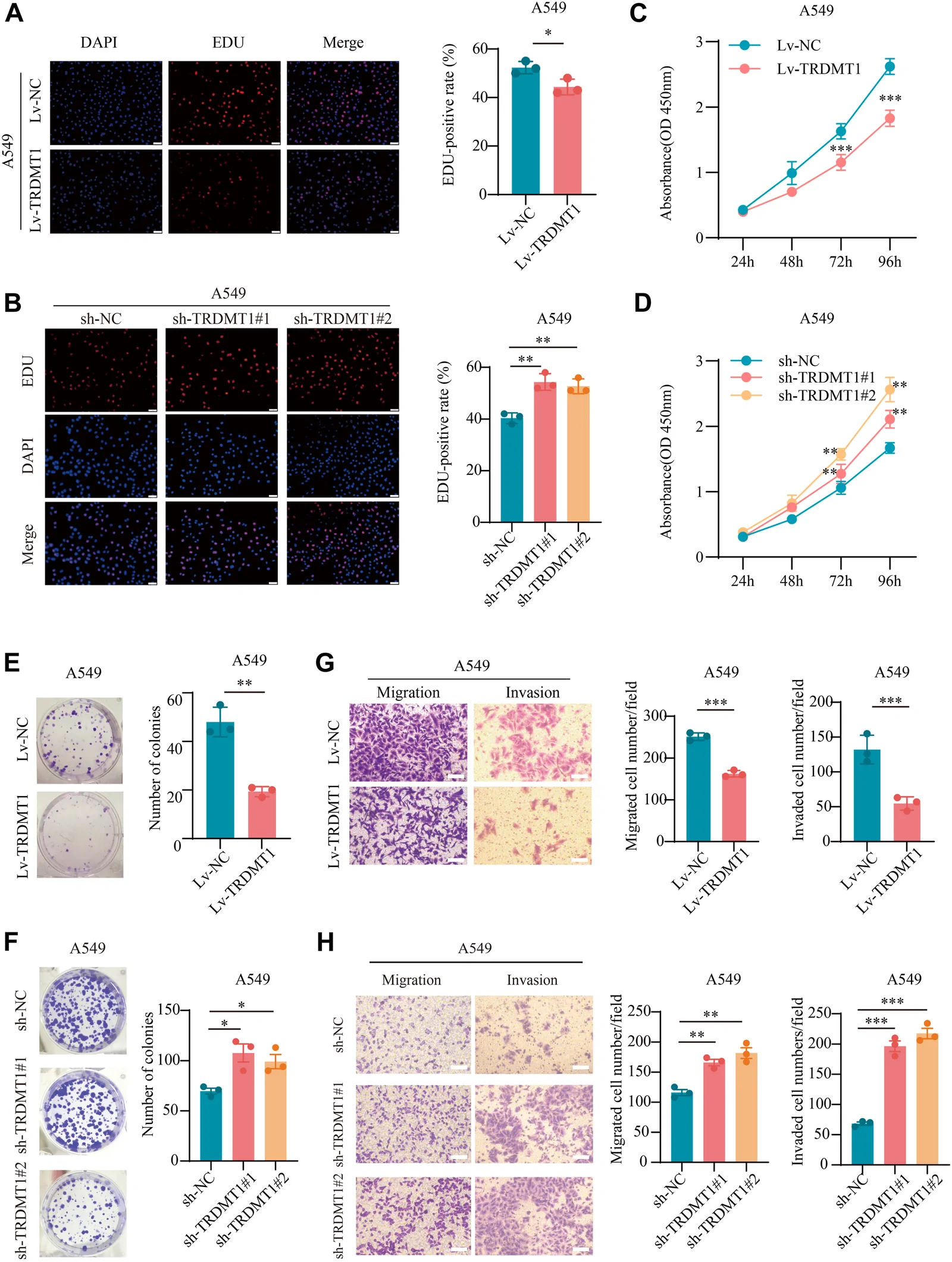
**TRDMT1 suppresses oncogenic phenotypes in LUAD cells *in vitro*.***A* and *B*, EdU assays evaluating the proliferation of A549 cells following *TRDMT1* overexpression (Lv-TRDMT1) (*A*) or knockdown (sh-TRDMT1#1 and #2) (*B*). Representative fluorescence images (*left*) and quantification of the EdU-positive cell rate (*right*) are shown. Scale bar = 100 μm. *C* and *D*, CCK-8 assays assessing the cell viability of A549 cells upon *TRDMT1* overexpression (*C*) or knockdown (*D*) over a 96-h period. *E* and *F*, colony formation assays illustrating the impact of *TRDMT1* overexpression (*E*) and knockdown (*F*) on the clonogenic ability of A549 cells. Representative well images and quantitative bar graphs are provided. *G* and *H*, Transwell migration (uncoated) and invasion (Matrigel-coated) assays evaluating the migratory and invasive capacities of A549 cells subjected to *TRDMT1* overexpression (*G*) or knockdown (*H*). Representative images (*left*) and quantification of migrated/invaded cells per field (*right*) are shown. Scale bar = 100 μm. Data are presented as mean ± SD from three independent experiments (*n* = 3). ∗*p* < 0.05, ∗∗*p* < 0.01, ∗∗∗*p* < 0.001, determined by two-tailed Student's *t* test (*A*, *E* and *G*) or one-way/two-way ANOVA followed by appropriate *post hoc* tests (*B*, *C*, *D*, *F* and *H*).

### Transcriptomic profiling identifies *TFRC* as a key target of TRDMT1 in the regulation of ferroptosis

Given the established functional significance of TRDMT1 in the pathogenesis of LUAD, we performed transcriptomic profiling via RNA sequencing (RNA-seq) in the validated stable *TRDMT1*-overexpressing H1299 cell line described above (*n* = 3 per group). This approach allowed us to systematically characterize *TRDMT1*-regulated transcriptional networks and signaling pathways, thereby elucidating its underlying mechanistic contributions to LUAD tumorigenesis (Fig. 4*A*). Comparative analysis of transcriptomes from *TRDMT1*-overexpressing and control cells identified 236 upregulated and 106 downregulated genes. Subsequent Gene Ontology (GO) and Kyoto Encyclopedia of Genes and Genomes (KEGG) enrichment analyses revealed several cancer-associated pathways modulated by TRDMT1, including DNA replication and cell cycle regulation (Fig. 4, *B* and *C*). Notably, several ferroptosis-associated genes were present among the differentially expressed genes. To investigate whether TRDMT1 acts as a potential regulator of ferroptosis, we performed an intersection analysis between *TRDMT1*-altered transcripts and a curated set of ferroptosis-related genes. This approach revealed that *TRDMT1* overexpression influenced the expression of 16 key ferroptosis-associated genes, as visually summarized in a heatmap comparing *TRDMT1*-overexpressing and control cells (Fig. 4, *D* and *E*).

**Figure 4:**
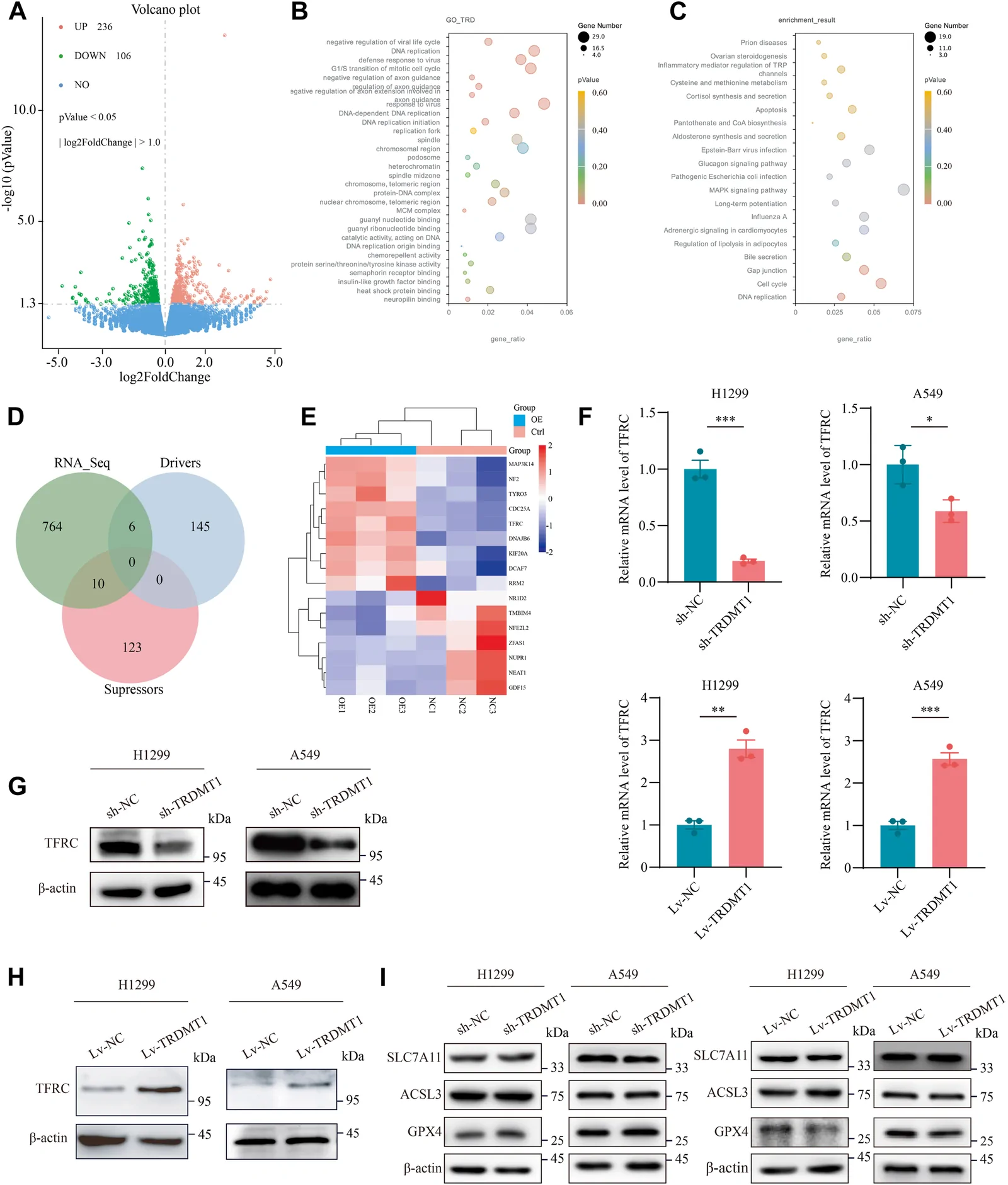
**Transcriptomic profiling identifies *TFRC* as a key target of *TRDMT1* in the regulation of ferroptosis.***A*, Volcano plot illustrating transcriptomic alterations in H1299 cells following *TRDMT1* overexpression. *Red* and *green dots* denote significantly upregulated and downregulated genes, respectively (|log2FoldChange| > 1.0 and *p* < 0.05). *B* and *C*, functional enrichment analysis of the differentially expressed genes (DEGs) *via* GO (*B*) and KEGG (*C*). *D*, Venn diagram showing the intersection of *TRDMT1*-regulated DEGs with ferroptosis drivers and suppressors from the FerrDb database, yielding 16 overlapping genes. *E*, heatmap depicting the expression patterns of the 16 overlapping ferroptosis-related genes across the overexpression (OE) and negative control (NC) groups. *F*, relative mRNA expression of *TFRC* measured by RT-qPCR in H1299 and A549 cells following *TRDMT1* knockdown (*top*) or overexpression (*bottom*). *G* and *H*, Western blot analysis of TFRC protein expression in H1299 and A549 cells following *TRDMT1* knockdown (*G*) and overexpression (*H*). *I*, Western blot detection of key ferroptosis marker proteins (SLC7A11, ACSL3, and GPX4) in H1299 and A549 cells following *TRDMT1* modulation. Data are presented as mean ± SD from at least three independent experiments (*n* = 3). ∗*p* < 0.05, ∗∗*p* < 0.01, ∗∗∗*p* < 0.001, determined by two-tailed Student's *t* test.

To functionally validate these findings, we focused on *TFRC*, one of the most prominently upregulated genes in the RNA-seq dataset. Consistent with the sequencing data, stable *TRDMT1* overexpression in H1299 and A549 cells increased *TFRC* mRNA levels, whereas *TRDMT1* knockdown resulted in a substantial reduction of *TFRC* transcripts (Fig. 4*F*). Importantly, this regulatory relationship was also evident at the protein level: TFRC abundance increased upon *TRDMT1* overexpression and decreased following *TRDMT1* knockdown (Fig. 4, *G* and *H*), confirming that TRDMT1 regulates TFRC at both the mRNA and protein levels.

Since TFRC serves as a key mediator of cellular iron uptake and iron metabolism homeostasis, we hypothesized that TRDMT1 may regulate ferroptosis via TFRC modulation. To test this hypothesis, we performed an initial assessment of the association between TRDMT1 and ferroptosis. *TRDMT1* was knocked down or overexpressed in A549 and H1299 cells, and changes in key ferroptosis-related proteins were examined (Fig. 4*I*). Results showed that *TRDMT1* knockdown consistently increased protein levels of the central ferroptosis defense factor GPX4 in both cell lines, while its overexpression reduced GPX4 expression. In contrast, alterations in SLC7A11 and ACSL3 were inconsistent across the two cell types, suggesting that they may be affected by cell type-specific mechanisms or other regulatory factors rather than serving as universal targets of TRDMT1. As *TFRC*—rather than *GPX4*—emerged from our RNA-seq intersection, these GPX4 changes most likely reflect a shifted ferroptotic set-point downstream of iron overload rather than direct m5C targeting.

### TRDMT1 disrupts iron homeostasis to potentiate ferroptosis

We next tested whether TRDMT1 modulates ferroptotic susceptibility. Using the FerroOrange probe, labile Fe^2+^ accumulation under Erastin or RSL3 was attenuated by *TRDMT1* knockdown (Fig. 5, *A* and *C*) but exacerbated by *TRDMT1* overexpression (Fig. 5, *B* and *D*). Consistently, *TRDMT1* depletion conferred resistance to both inducers, whereas its overexpression heightened sensitivity and accelerated cell death (Fig. 5, *E* and *F*). To directly evaluate ferroptotic oxidative stress, we utilized the C11-BODIPY fluorescent sensor to quantify lipid ROS levels *via* flow cytometry (Fig. 5*G*). We observed that *TRDMT1* knockdown markedly suppressed Erastin-induced lipid ROS accumulation, a finding corroborated by our MDA assay results (Fig. 5*H*). Collectively, these data provide robust evidence that TRDMT1 amplifies lipid peroxidation, the critical executioner step of ferroptosis. Thus, TRDMT1 amplifies ferroptosis by promoting Fe^2+^-driven lipid peroxidation in LUAD.

**Figure 5:**
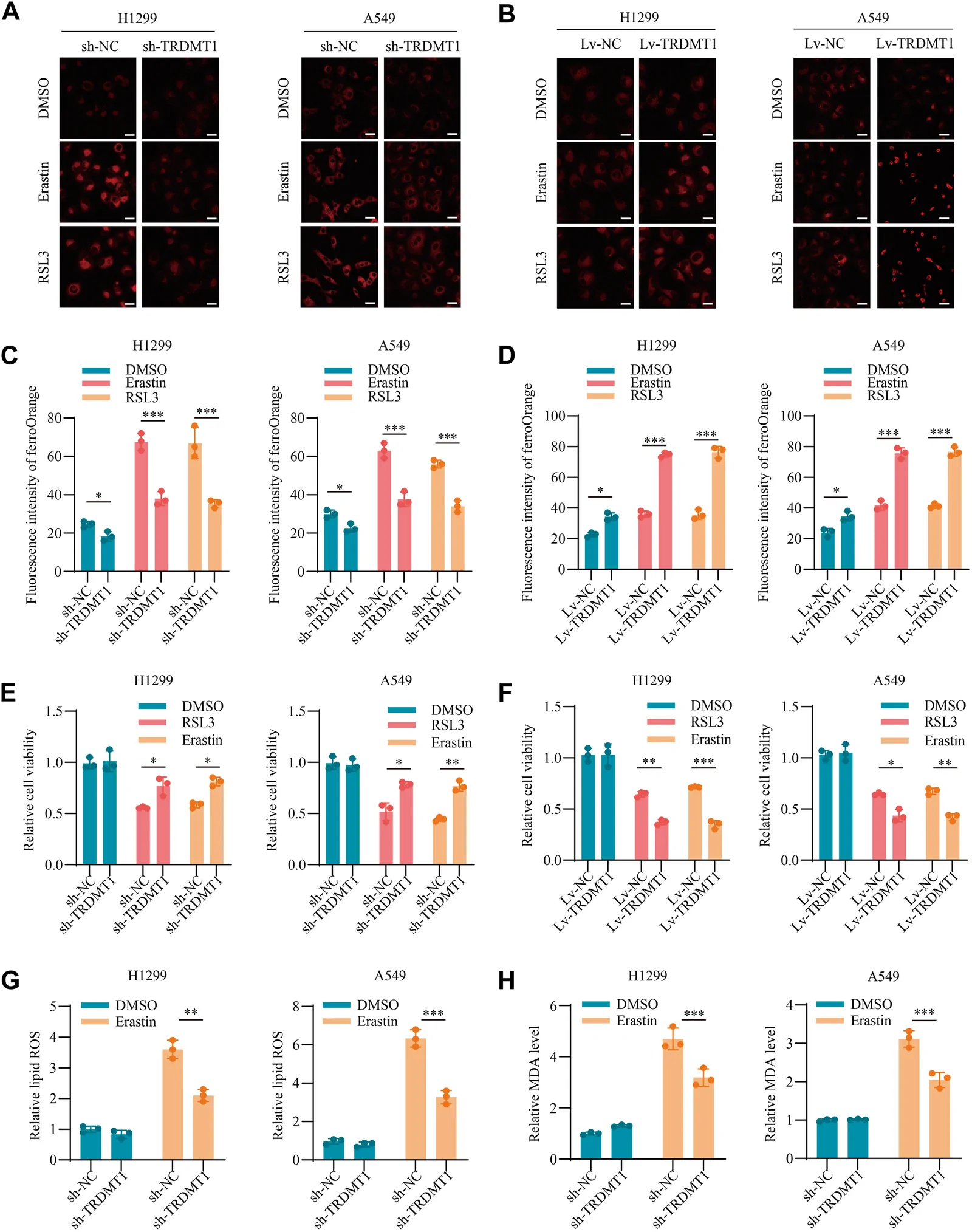
**TRDMT1 disrupts iron homeostasis to potentiate ferroptosis.***A* and *B*, representative images of intracellular Fe^2+^ levels detected via the FerroOrange fluorescent probe in H1299 and A549 cells following *TRDMT1* knockdown (*A*) or overexpression (*B*), treated with DMSO, Erastin, or RSL3. Scale bar = 20 μm. *C* and *D*, Quantitative analysis of the relative fluorescence intensity from panels (*A*) and (*B*), respectively. *E* and *F*, relative cell viability evaluated by CCK-8 assay in H1299 and A549 cells following *TRDMT1* knockdown (*E*) or overexpression (*F*), subjected to DMSO, RSL3, or Erastin treatment. *G*, quantitative analysis of lipid ROS accumulation measured by C11-BODIPY staining in *TRDMT1*-knockdown H1299 and A549 cells treated with DMSO or Erastin. *H*, relative malondialdehyde (MDA) levels determined in *TRDMT1*-knockdown H1299 and A549 cells following DMSO or Erastin treatment. Data are presented as mean ± SD from three independent experiments (*n* = 3). ∗*p* < 0.05, ∗∗*p* < 0.01, ∗∗∗*p* < 0.001, determined by two-way ANOVA followed by appropriate *post hoc* tests.

To confirm that this death was specifically ferroptotic, we performed exclusion and rescue assays. *TRDMT1* knockdown altered neither the executioner proteins of necroptosis (pMLKL/tMLKL), pyroptosis (GSDME), nor apoptosis (CASP3) (Fig. S4, *A–C*), excluding these pathways. The heightened Erastin cytotoxicity in *TRDMT1*-overexpressing cells was fully rescued by Ferrostatin-1 (Fig. S4*D*). Moreover, *TRDMT1* knockdown protected cells against death and MDA accumulation induced by acrolein, a polyamine-derived ferroptosis inducer (36) (Fig. S4, *E* and *F*). Together, these data confirm that TRDMT1 specifically amplifies ferroptosis rather than broadly sensitizing cells to other death modalities.

### TRDMT1 enhances TFRC mRNA stability through m5C-dependent modification

Because TRDMT1 increases mature *TFRC* mRNA without affecting its pre-mRNA (Figs. 6*A* and S4*G*), this regulation likely occur post-transcriptionally. To further investigate whether TRDMT1 modulates TFRC expression at the protein stability level, cells were treated with cycloheximide (CHX). Western blot analysis showed no notable difference in TFRC protein half-life between control and *TRDMT1*-overexpressing groups (Fig. 6, *B* and *C*), implying that TRDMT1 likely governs TFRC expression via RNA modification.

**Figure 6:**
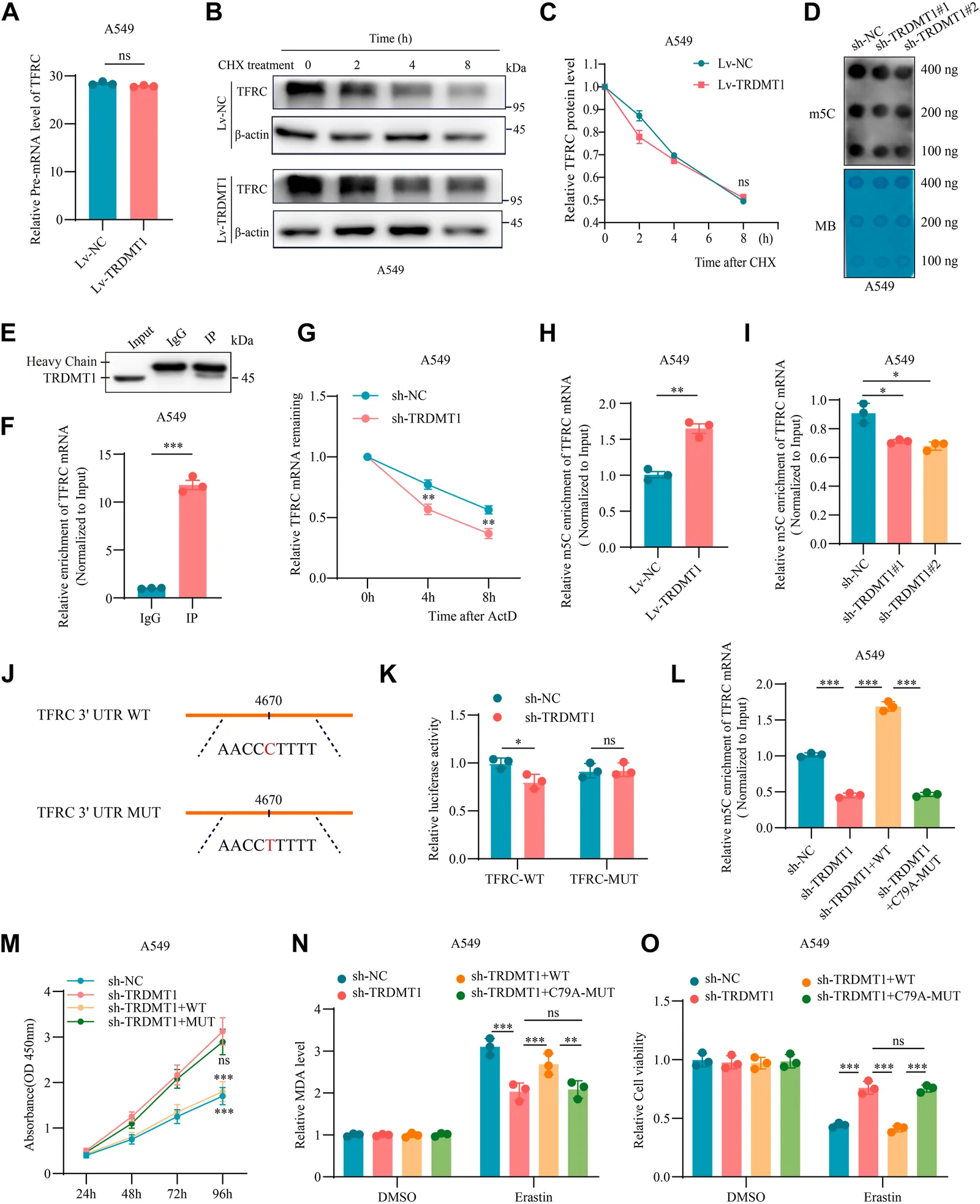
**TRDMT1 enhances *TFRC* mRNA stability through m5C-dependent modification.***A*, relative pre-mRNA levels of *TFRC* in A549 cells following *TRDMT1* overexpression, assessed by RT-qPCR. *B* and *C*, Western blot analysis (*B*) and corresponding quantification (*C*) of TFRC protein degradation in A549 cells treated with cycloheximide (CHX) for the indicated times (0, 2, 4, 8 h). *D*, Dot blot assay evaluating global m5C RNA methylation levels in A549 cells following *TRDMT1* knockdown. Methylene *blue* (MB) staining served as a loading control. *E* and *F*, RNA immunoprecipitation (RIP) assay in A549 cells. Western blot analysis of TRDMT1 protein in the Input, IgG, and IP samples (*E*), and RT-qPCR quantification of *TFRC* mRNA enrichment in the TRDMT1 immunoprecipitates relative to the IgG control (*F*). *G*, mRNA stability assay. A549 cells (sh-NC and sh-TRDMT1) were treated with Actinomycin D (ActD) to arrest transcription, and *TFRC* mRNA decay rates were measured by RT-qPCR at 0, 4, and 8 h. *H* and *I*, methylated RNA immunoprecipitation (MeRIP)-qPCR assay evaluating m5C enrichment on *TFRC* mRNA in A549 cells following *TRDMT1* overexpression (*H*) or knockdown (*I*). *J*, schematic representation of the dual-luciferase reporter constructs containing the wild-type (WT) or mutant (MUT) *TFRC* 3′UTR encompassing the candidate m5C modification site C4670. *K*, relative luciferase activity in A549 cells co-transfected with WT or MUT *TFRC* reporter plasmids and *TRDMT1* knockdown vectors. *L*, MeRIP-qPCR assay assessing m5C enrichment on *TFRC* mRNA in A549 cells subjected to *TRDMT1* knockdown, followed by rescue with wild-type TRDMT1 (WT) or a catalytically inactive mutant (C79A-MUT). *M–O*, rescue experiments evaluating the functional dependence of TRDMT1 on its catalytic activity. CCK-8 proliferation assay (*M*), relative MDA levels (*N*), and relative cell viability (*O*) in the indicated A549 cell groups treated with DMSO or Erastin. Data are presented as mean ± SD from three independent experiments (*n* = 3). ns, not significant, ∗*p* < 0.05, ∗∗*p* < 0.01, ∗∗∗*p* < 0.001, determined by two-tailed Student's *t* test (*A*, *F*, *H* and *I*), one-way ANOVA (*L*), or two-way ANOVA (*C*, *G*, *K*, *M*, *N* and *O*).

Given that TRDMT1 is an established methyltransferase, we sought to determine whether it functions as an m5C methyltransferase in this context. Dot blot assays demonstrated that *TRDMT1* knockdown resulted in a marked reduction in global m5C levels (Fig. 6*D*), whereas its overexpression led to an elevation (Fig. S4*H*), further supporting its methyltransferase activity. We next performed RNA immunoprecipitation (RIP) using a TRDMT1-specific antibody to examine potential direct interaction between TRDMT1 and *TFRC* mRNA. Following verification of successful TRDMT1 immunoprecipitation *via* Western blotting (Figs. 6*E* and S4*I*), RT-qPCR analysis of the immunoprecipitated RNA demonstrated significant enrichment of *TFRC* mRNA in TRDMT1 pull-down samples relative to IgG controls (Figs. 6*F* and S4*J*), supporting an association between TRDMT1 and *TFRC* mRNA.

To elucidate the mechanism by which TRDMT1-mediated m5C methylation influences *TFRC* mRNA expression, Actinomycin D was administered to *TRDMT1*-knockdown A549 and H1299 cells. TFRC mRNA exhibited accelerated decay in *TRDMT1*-depleted cells (Figs. 6*G* and S4*K*), indicating that TRDMT1 enhances *TFRC* mRNA stability in LUAD cells. Subsequently, m5C-specific methylated RNA immunoprecipitation (MeRIP) coupled with RT-qPCR was employed to assess TRDMT1-dependent m5C modification on *TFRC* mRNA. Overexpression of *TRDMT1* significantly augmented m5C modification levels on *TFRC* transcripts (Figs. 6*H* and S4*L*), while *TRDMT1* knockdown substantially diminished them (Figs. 6*I* and S4*M*), confirming that TRDMT1 catalyzes m5C methylation on *TFRC* mRNA.

To establish that TRDMT1 regulates *TFRC* *mRNA* stability via direct m5C modification, we conducted a mutational analysis of candidate methylation sites. To identify functionally relevant and reported m5C modifications on the *TFRC* transcript, we directly interrogated the RMVar database. Although several mapped sites were distributed across the 5′ UTR and coding sequence (CDS), we focused on the 3' UTR—the canonical regulatory hub for RNA stability—to avoid confounding amino acid substitutions or translation-initiation effects. This identified the cytosine at position 4670 (C4670) as the target for site-directed mutagenesis and dual-luciferase reporter assays. We constructed luciferase reporter plasmids containing either the wild-type *TFRC* 3′ UTR (WT) or a mutant sequence (MUT) in which the targeted cytosine was substituted (Fig. 6*J*). Dual-luciferase reporter assays revealed that *TRDMT1* knockdown significantly repressed the luciferase activity of the WT reporter. In contrast, the activity of the MUT reporter remained unaffected by *TRDMT1* depletion (Fig. 6*K*).

To confirm that the methyltransferase catalytic activity of TRDMT1 is required for TFRC regulation and ferroptosis sensitivity, we performed rescue experiments using a catalytically dead mutant. Specifically, we re-introduced either wild-type TRDMT1 (WT) or the catalytically inactive mutant (C79A-MUT) into *TRDMT1*-knockdown cells. The C79A substitution is a widely established strategy to abrogate the m5C methyltransferase activity of TRDMT1 (37, 38). MeRIP-qPCR analysis verified that ectopic expression of TRDMT1-WT successfully restored m5C enrichment on *TFRC* mRNA, whereas the C79A-MUT construct failed to do so (Fig. 6*L*), confirming that TRDMT1 mediates TFRC methylation in an enzymatic activity-dependent manner. Furthermore, phenotypic rescue assays demonstrated that wild-type TRDMT1 effectively restored both the tumor-suppressive and pro-ferroptotic phenotypes in *TRDMT1*-depleted cells (Fig. 6, *M–O*), whereas the catalytically dead C79A mutant failed to do so. This establishes that the biological functions of TRDMT1 strictly depend on its m5C methyltransferase activity.

### TFRC is downregulated in LUAD, and its overexpression suppresses LUAD tumorigenesis

After identifying TFRC as a direct downstream target of TRDMT1, we assessed its clinical relevance in LUAD, revealing that *TFRC* was significantly downregulated in clinical LUAD specimens (Fig. 7*A*). Subsequent survival analyses corroborated this trend, showing that low *TFRC* levels correlate with poorer overall survival and unfavorable patient prognosis (Fig. 7, *B* and *C*).

**Figure 7:**
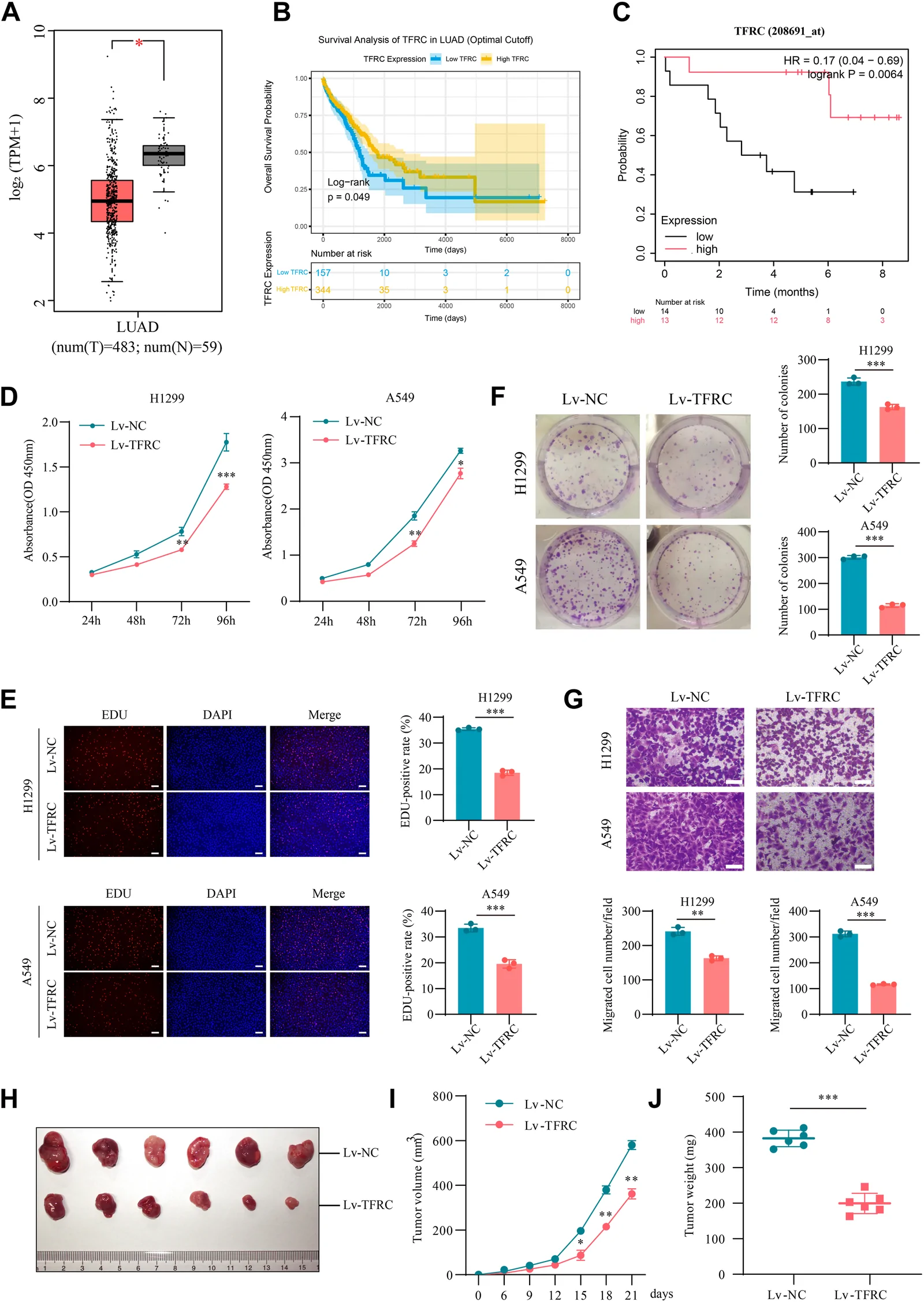
**TFRC is downregulated in LUAD, and its overexpression suppresses LUAD tumorigenesis.***A*, box plot showing *TFRC* mRNA expression in LUAD (Tumor = 483) and normal tissues (Normal = 59) from GEPIA. *B* and *C*, Kaplan–Meier overall survival curves of LUAD patients stratified by high and low *TFRC* expression levels based on the TCGA cohort (*B*) and Kaplan–Meier Plotter (*C*). *D*, CCK-8 assay evaluating the cell viability of H1299 and A549 cells over a 96-h period following *TFRC* overexpression (Lv-TFRC) or empty vector control (Lv-NC). *E*, EdU assay assessing the proliferation of H1299 and A549 cells following *TFRC* overexpression. Representative fluorescence images (*left*) and quantification of the EdU-positive cell rate (*right*) are shown. Scale bar = 100 μm. *F*, colony formation assay in H1299 and A549 cells following *TFRC* overexpression. Representative images of cell colonies (*left*) and quantitative bar graphs (*right*). *G*, Transwell migration assay evaluating the migratory capacity of H1299 and A549 cells following *TFRC* overexpression. Representative images (*left*) and quantification of migrated cells per field (*right*). Scale bar = 100 μm. *H–J*, *in vivo* xenograft tumor model. Representative images of the excised tumors from the Lv-NC and Lv-TFRC groups (*n* = 6 mice per group) (*H*), along with the corresponding tumor growth curves measuring volume over 21 days (*I*) and final tumor weights (*J*). Data are mean ± SD from three independent experiments (*D–G*) or from six mice per group (H–J). ∗*p* < 0.05, ∗∗*p* < 0.01, ∗∗∗*p* < 0.001, determined by log-rank test (*B* and *C*), two-tailed Student's *t* test (*A*, *E*, *F*, *G* and *J*), or two-way ANOVA (*D* and *I*).

Given these clinical correlations, we next investigated the functional role of TFRC in LUAD progression. To determine whether TFRC itself affects LUAD malignant behavior, we stably overexpressed *TFRC* in A549 and H1299 cell lines, confirming the overexpression efficiency *via* Western blotting (Fig. S2*E*). CCK-8 assays showed that elevated TFRC significantly impaired cell viability (Fig. 7*D*). This growth defect was further evidenced by a marked reduction in EdU-positive proliferating cells (Fig. 7*E*) and compromised long-term colony-forming capacity (Fig. 7*F*) following TFRC induction. Additionally, Transwell assays demonstrated a severe impairment in the migratory potential of *TFRC*-overexpressing cells (Fig. 7*G*).

To extend these *in vitro* findings, we employed a subcutaneous xenograft mouse model. Mice implanted with *TFRC*-overexpressing cells developed tumors that were significantly smaller in both volume and weight compared to control xenografts (Fig. 7, *H*–*J*).

### TRDMT1 suppresses tumor progression and promotes ferroptosis via TFRC *in vitro* and *in vivo*

Given the limited mechanistic insight into the TRDMT1-mediated regulation of TFRC in lung cancer, we performed *TFRC* reconstitution experiments in *TRDMT1*-knockdown cells to delineate their functional interplay (Fig. 8*A*). EdU incorporation assays revealed that *TRDMT1* depletion promoted LUAD cell proliferation, an effect that was significantly attenuated upon *TFRC* overexpression (Fig. 8*B*). Similarly, Transwell migration assays indicated that the enhanced migratory capacity resulting from *TRDMT1* knockdown was effectively reversed by *TFRC* reconstitution in LUAD cells (Fig. 8*C*). Under Erastin-induced ferroptotic stress, *TFRC* overexpression restored the level of lipid peroxidation that had been suppressed by *TRDMT1* knockdown (Fig. 8*D*), indicating that TRDMT1 promotes ferroptosis by enhancing TFRC-mediated lipid peroxidation.

**Figure 8:**
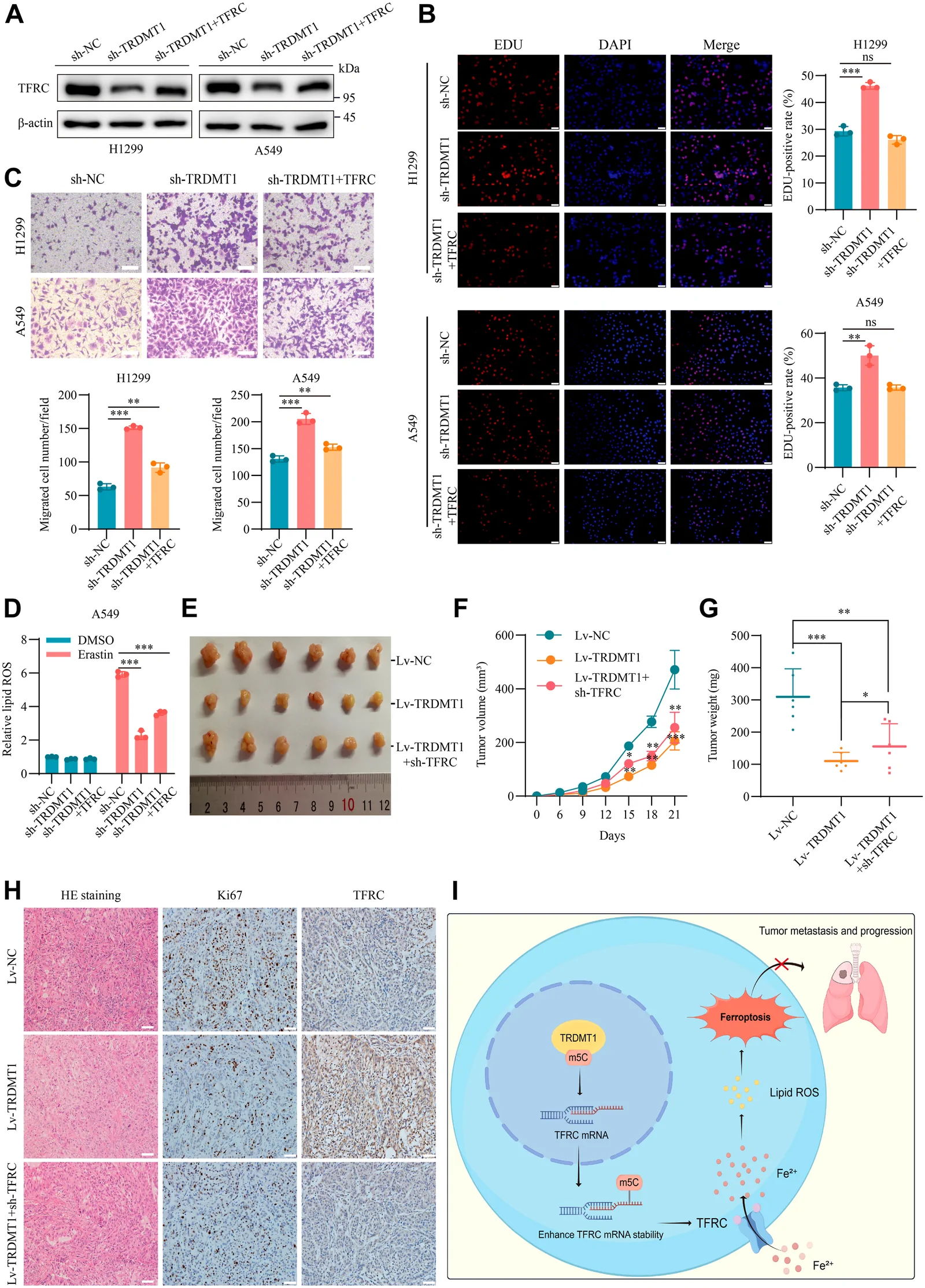
**TRDMT1 suppresses tumor progression and promotes ferroptosis via TFRC *in vitro* and *in vivo*.***A*, Western blot analysis of TFRC protein expression in H1299 and A549 cells following *TRDMT1* knockdown (sh-TRDMT1) alone or in combination with *TFRC* overexpression (sh-TRDMT1+TFRC). *B*, EdU assay evaluating the proliferation of H1299 and A549 cells across the indicated groups. Representative fluorescence images (*left*) and quantification of the EdU-positive cell rate (*right*). Scale bar = 100 μm. *C*, Transwell migration assay assessing the migratory capacity of H1299 and A549 cells in the indicated groups. Representative images (*top*) and quantification of migrated cells per field (*bottom*). Scale bar = 100 μm. *D*, relative lipid ROS levels in A549 cells quantified by flow cytometry using C11-BODIPY staining in the indicated groups treated with DMSO or Erastin. *E–G*, *in vivo* xenograft tumor model evaluating the rescue effect of *TFRC* knockdown (sh-TFRC) on *TRDMT1* overexpression (Lv-TRDMT1). Representative photographs of excised tumors at day 21 post-implantation (*E*), tumor growth curves measuring volume over time (*F*), and final tumor weights at the experimental endpoint (*G*). (*n* = 6 mice per group). *H*, representative images of H&E, Ki-67, and TFRC immunohistochemical (IHC) staining in the excised tumor sections. Scale bar = 50 μm. *I*, schematic model illustrating that TRDMT1 enhances *TFRC* mRNA stability *via* m5C modification, thereby promoting ferroptosis and suppressing lung cancer progression and metastasis. Data are mean ± SD from three independent experiments or from six mice per group (*E–G*). ns, not significant, ∗*p* < 0.05, ∗∗*p* < 0.01, ∗∗∗*p* < 0.001, determined by one-way ANOVA (*B*, *C* and *G*) or two-way ANOVA (*D* and *F*).

To extend these findings *in vivo*, we established stable cell lines overexpressing *TRDMT1* with concomitant *TFRC* knockdown and evaluated their tumorigenic potential using a subcutaneous xenograft model in nude mice. Tumor growth was monitored until the experimental endpoint, at which point tumors were excised and weighed (Fig. 8, *E*–*G*). As expected, compared with the *TRDMT1*-overexpressing group, loss of *TFRC* partially reversed the tumor-suppressive effect of TRDMT1, leading to a significant recovery in tumor growth metrics, including both volume and weight.

IHC staining for Ki-67 showed fewer Ki-67-positive nuclei in *TRDMT1*-overexpressing tumors, and this reduction was attenuated by concurrent *TFRC* knockdown (Fig. 8*H*). Furthermore, IHC analysis confirmed elevated TFRC expression in *TRDMT1*-overexpressing tumors, and that TFRC expression was notably downregulated in the group with combined *TRDMT1* overexpression and *TFRC* knockdown, verifying effective TFRC knockdown *in vivo* (Fig. 8*H*). Collectively, these *in vivo* rescue data are consistent with our *in vitro* observations, indicating that TRDMT1 suppresses tumor growth largely through TFRC-dependent ferroptosis (Fig. 8*I*).

## Discussion

Lung cancer continues to pose a significant global health burden due to therapeutic resistance and tumor heterogeneity (39). In this context, m5C RNA modifications have emerged as critical post-transcriptional regulators of oncogenesis (40, 41). While TRDMT1, an early-identified m5C "writer," has been implicated in malignancies such as glioblastoma and osteosarcoma (24, 42), its specific mechanistic contributions to LUAD have remained largely unexplored. In the present study, we identify TRDMT1 as a robust tumor suppressor and a favorable prognostic factor in LUAD. Rather than solely regulating classical proliferative pathways, our data reveal that TRDMT1 exerts its anti-tumorigenic effects by fundamentally rewiring iron metabolism to hypersensitize cancer cells to ferroptosis.

Ferroptosis, an iron-dependent form of cell death driven by lipid peroxidation (43), is tightly controlled by the system Xc^−^–GSH–GPX4 axis (44). While emerging evidence links m5C regulators, such as NSUN2, to drug resistance and redox homeostasis in lung cancer (45), the involvement of TRDMT1 has remained undefined. Our findings bridge this gap by demonstrating that TRDMT1 acts as a pivotal epigenetic rheostat for ferroptosis. By post-transcriptionally stabilizing *TFRC* mRNA in an m5C-dependent manner, TRDMT1 drives intracellular Fe^2+^ accumulation and synergizes with classical ferroptosis inducers (Erastin/RSL3) to execute lipid peroxidation and cell death. This positions RNA m5C methylation as an upstream vulnerability of the ferroptotic cascade.

TFRC, a central mediator of cellular iron uptake, is increasingly recognized as a key regulator of ferroptosis susceptibility across various pathological contexts (46, 47, 48). Canonically, *TFRC* is regarded as a pro-tumorigenic gene that fuels the "iron addiction" of cancer cells to facilitate tumor progression (49). However, recent seminal studies have established a context-dependent paradigm: TFRC acts as an obligate driver of ferroptosis (50). While moderate expression supports basal tumor growth, this excessive iron dependency exposes a critical vulnerability to iron-dependent death (51). This duality is evident even within lung cancer itself, as documented by independent studies. On one hand, TFRC has been reported to play its classical pro-tumorigenic role: Wu *et al*. showed that the transferrin receptor is expressed in most lung cancers, is associated with poor prognosis in NSCLC, and that blocking it suppresses lung adenocarcinoma cell proliferation by attenuating oncogenic KRAS signaling (52). On the other hand, TFRC also acts on the ferroptosis-driving, tumor-suppressive side: Zhang *et al*. reported that *TFRC* is downregulated in lung cancer tissue and that restoring *TFRC*—by relieving METTL3-mediated m6A destabilization of *TFRC* mRNA—enhances ferroptosis sensitivity, whereas METTL3 attenuation suppresses tumor growth (53). Thus, even within lung cancer, independent groups have found TFRC to act as either an oncogenic or a tumor-suppressive factor depending on the iron/redox context—precisely the duality we observe. This dual nature contextualizes a seemingly paradoxical aspect of our findings: while classically pro-oncogenic, *TFRC* was downregulated in our LUAD cohort, low *TFRC* expression predicted poorer survival, and TRDMT1-driven TFRC upregulation suppressed tumor growth. We reconcile these observations through a biphasic, context-dependent model of iron biology. Under basal conditions, moderate iron import supports proliferative metabolism (48); however, once iron acquisition is driven beyond a homeostatic threshold—as occurs upon TRDMT1-mediated *TFRC* mRNA stabilization—the resulting labile Fe^2+^ overload drives lethal Fenton chemistry, lowers the ferroptotic threshold, and tips cells toward iron-catalyzed death (47, 49). This framework explains why *TFRC* overexpression alone restrained LUAD growth (Fig. 7), whereas exogenous ferroptotic stress markedly amplified this effect (Figure 5, Figure 8). Thus, TFRC acts as a metabolic rheostat whose net effect on LUAD is dictated by the magnitude of iron loading. Supported by our rescue assays showing that *TFRC* downregulation reverses TRDMT1-induced ferroptosis, our results delineate a model wherein TRDMT1 enhances *TFRC* mRNA stability to suppress LUAD development, though further mechanistic details remain to be fully elucidated.

In summary, our findings illuminate an epigenetic-metabolic circuit wherein the TRDMT1–TFRC axis sensitizes LUAD cells to ferroptosis (Fig. 8*I*). This nominates TRDMT1 restoration as a promising therapeutic strategy to trigger ferroptotic cell death in resistant tumors. However, translating this axis into clinical application faces several challenges. First, the mechanistic landscape remains incomplete. It is still unclear whether specific m5C "reader" proteins, such as ALYREF or YBX1, are required to recognize the m5C modification on *TFRC* mRNA and mediate its mRNA stabilization. Second, our current experimental systems are inadequate for evaluating the long-term safety of sustained TRDMT1 activation. The generation of conditional or inducible TRDMT1 overexpression mouse models will be essential to assess potential systemic toxicities associated with chronic pathway modulation *in vivo*. Furthermore, clinical translation must address issues of tumor heterogeneity, potential acquired resistance, and risks of off-target effects or normal tissue damage resulting from systemic ferroptosis induction. We also note that the four-gene m5C prognostic signature, although capturing the biological relevance of m5C dysregulation in LUAD, achieved only modest discrimination (time-dependent AUCs 0.599–0.647). It is therefore intended as a hypothesis-generating, biology-oriented classifier rather than a standalone clinical predictor, and would require larger multi-center validation and integration with established clinicopathological variables before any prognostic use. Future studies should focus on delineating the full regulatory network underlying this axis and validating its long-term safety profile. The development of sophisticated targeting strategies in physiologically relevant models will be critical to advancing this pathway toward clinical application.

## Experimental procedures

### Cell culture and generation of stable cell lines

The human lung adenocarcinoma (LUAD) cell line A549 and the representative non-small cell lung cancer (NSCLC) cell line H1299—widely utilized together to robustly model the biological spectrum of LUAD/NSCLC— were procured from Procell Life Science & Technology Co., Ltd . All cell lines were authenticated by STR profiling and routinely tested negative for mycoplasma. Cells were maintained in RPMI-1640 or DMEM medium (Gibco), supplemented with 10% fetal bovine serum (FBS; LONSERA) and 1% penicillin-streptomycin (Solarbio), at 37 °C in a humidified 5% CO_2_ incubator.

To generate stable knockdown or overexpression models, cells were transduced with lentiviral particles carrying short hairpin RNA (shRNA) targeting *TRDMT1* (sh-TRDMT1#1, sh-TRDMT1#2) or *TFRC* (sh-TFRC), full-length *TRDMT1/TFRC* overexpression constructs (Lv-TRDMT1, Lv-TFRC), or their corresponding controls (sh-NC, Lv-NC), at a multiplicity of infection (MOI) of 50 in the presence of 8 μg/ml Polybrene (Sigma-Aldrich). Stable polyclonal populations were selected and maintained using 2 μg/ml puromycin for 21 days.

### Transient transfection and catalytic mutant generation

Cells were seeded in six-well plates and transfected at 70 to 80% confluence using Lipofectamine 3000 (Invitrogen, USA), adhering to the manufacturer's protocols. To investigate the m5C catalytic dependence of *TRDMT1*, wild-type (WT) *TRDMT1* and catalytically inactive mutant *TRDMT1* (C79A-MUT) plasmids were synthesized (MiaolingBio) and transfected into cells. Transfection efficiency was validated at the protein and RNA levels by Western blot and RT-qPCR 48 to 72 h post-transfection.

### Western blot analysis

Whole-cell lysates were extracted using RIPA buffer (Yeasen) supplemented with protease and phosphatase inhibitors (Beyotime). Protein concentrations were determined using a BCA assay kit (Leagene). Equal amounts of protein (20–30 μg) were denatured, separated by SDS-PAGE, and transferred to PVDF membranes (Millipore). After blocking with 5% non-fat milk or BSA for 1 h at room temperature, membranes were incubated overnight at 4 °C with specific primary antibodies. The primary antibodies used included TRDMT1 (19221-1-AP, Proteintech), TFRC (CY6618, Abways), and ferroptosis markers SLC7A11 (26864-1-AP, Proteintech), GPX4 (CY6959, Abways), and ACSL3 (20710-1-AP, Proteintech). Furthermore, antibodies against other cell death pathway markers were utilized: pMLKL (ab271971, Abcam) and tMLKL (ab184718, Abcam) for necroptosis, GSDME (ab219800, Abcam) for pyroptosis, and CASP3 (19677-1-AP, Proteintech) for apoptosis. β-Actin (81115-1-RR, Proteintech) served as the loading control. Following incubation with appropriate HRP-conjugated secondary antibodies, protein bands were visualized with an ECL detection reagent (Beyotime) using a ChemiDoc Imaging System (BioRad). Band intensities were quantified using ImageJ software (NIH) and normalized to β-Actin.

### Single-cell RNA-seq analysis

Raw count matrices of GSE131907 and GSE189357 were analyzed with scanpy (v1.9.3), anndata (v0.7.8), leidenalg (v0.10.1) and louvain (v0.8.1), and Seurat. Raw count matrices were preprocessed with DecontX (celda, version 1.6.1) for background ambient RNA noise removal, and scrublet (version 0.2.3) was used to detect and eliminate doublet cells. Cells with nFeature_RNA between 200 and 5000, total UMI counts between 0 and 30,000, ribosomal gene percentage < 50%, and mitochondrial gene percentage under the Singleron built-in threshold were retained (84,338 cells). Data were normalized *via* LogNormalize transformation, and Harmony (version 0.0.6, Sample ID as batch source) was used for batch-effect correction. The top 2000 highly variable genes were selected for dimensional reduction, and PCA dimensions followed the platform default setting. Clustering was performed at Leiden resolution 0.1 and visualized by UMAP; cell types were annotated automatically by CellID (version 0.1.0) algorithm relying on canonical cell markers, with *Homo sapiens* lung tissue as reference. Tumor epithelial cells (*n* = 11,374) were reclustered into six subgroups. Pseudotime trajectory was inferred with Monocle (v3.2.0). C1 and C5 signature scores were calculated with AddModuleScore and patients were dichotomized at median signature score for Kaplan–Meier analysis.

### RNA extraction, RT-qPCR, and mRNA/protein stability assays

Total RNA was isolated using TRIzol reagent (Sigma). cDNA was synthesized from 1 μg of total RNA using the Hifair III first Strand cDNA Synthesis SuperMix (Yeasen). RT-qPCR was performed in triplicate using SYBR Green Master Mix (Vazyme) on a QuantStudio Dx instrument (Life Technologies). *ACTB* or *GAPDH* served as endogenous controls for normalization using the 2^-ΔΔCT^ method.

### mRNA stability assay

To evaluate mRNA stability, stably transfected cells were treated with the transcription inhibitor Actinomycin D (ActD, 5 μg/ml). Total RNA was harvested at designated time points (0 h, 4 h, 8 h). Transcript levels of *TFRC* were quantified via RT-qPCR and normalized to the 0 h time point (set as 1) to determine the relative remaining mRNA levels over time.

### Protein stability assay

To assess TFRC protein degradation, the corresponding cells were treated with the protein synthesis inhibitor Cycloheximide (CHX, 50 μg/ml). Total protein was collected at 0 h, 2 h, 4 h, and 8 h post-treatment, and TFRC protein levels were analyzed by Western blot as described above. Protein band intensities were quantified and normalized to the 0 h control to evaluate the relative degradation rate.

### Immunohistochemistry (IHC) of clinical tissues and xenografts

Formalin-fixed, paraffin-embedded tissue sections from LUAD patients or xenograft mouse models were deparaffinized, rehydrated, and subjected to heat-induced antigen retrieval. Endogenous peroxidase activity was quenched with 3% H_2_O_2_. Sections were blocked with goat serum (Servicebio) and incubated overnight at 4 °C with primary antibodies against TRDMT1 (19221-1-AP, Proteintech), TFRC (CY8260, Abways), or Ki-67 (CY5542, Abways). Immunoreactivity was detected using a biotin-labeled secondary antibody and a DAB substrate kit (Servicebio). Sections were counterstained with hematoxylin.

As established to eliminate observer bias, a semiquantitative evaluation was performed double-blinded. IHC was scored using the immunoreactive score (IRS) system (proportion of positive cells: 0–4; multiplied by staining intensity: 0–3; total range 0–12). Slides were independently evaluated by two pathologists blinded to sample group allocations, clinical data, and corresponding transcriptomic profiles. Final scores were assigned by consensus upon mutual review.

### Cell proliferation, viability, and ferroptosis induction assays

#### Colony formation assay

Cells were seeded at 800 cells per well in 6-well plates and cultured for 14 days. Resulting colonies were fixed with methanol, stained with 0.1% crystal violet, and manually counted (colonies >50 cells).

#### Ferroptosis induction

Cells were treated with Erastin (10 μM, 12 h; S7242, Selleck), RSL3 (4 μM, 12 h; S8155, Selleck), Acrolein (100 μM, 10 h; 30646, Restek), or DMSO as vehicle control. For rescue experiments, cells were pre-treated with Ferrostatin-1 (10 μM; HY-100579, MCE) for 48 h.

#### CCK-8 assay

Cells were plated in 96-well plates at a density of 1500 cells/well. Cell viability was evaluated using the Cell Counting Kit-8 (40203ES60; Yeasen). For cell growth curves, 10 μl of CCK-8 reagent was added to each well at 24, 48, 72, and 96 h after seeding. For ferroptosis viability assays, 10 μl of CCK-8 reagent was added 12 h after the respective drug treatments. Following the addition of the reagent, plates were incubated for 2 h at 37 °C, and the absorbance at 450 nm was measured using a microplate reader.

#### EdU assay

Cell proliferation was assessed using the Cell-Light EdU Apollo567 In Vitro Kit (RiboBio). Cells were incubated with EdU, fixed, permeabilized, and stained with Apollo dye and DAPI. EdU-positive rates were calculated from at least three randomly selected fields under a fluorescence microscope (Olympus).

### Cell migration and invasion assays

Cell migratory and invasive capacities were evaluated using 24-well Transwell chambers equipped with 8-μm pore size polycarbonate membrane inserts (Corning). For the invasion assays, the upper surfaces of the membranes were pre-coated with Matrigel (Corning) to simulate the extracellular matrix, whereas the migration assays utilized uncoated inserts. Cells were harvested, resuspended in a serum-free medium, and seeded into the upper chambers at a density of 1 × 10^5^ cells per well in 200 μl. The lower chambers were filled with 600 μl of complete medium supplemented with 20% FBS to serve as a chemoattractant. Following a 24-h incubation at 37 °C, the non-migrating cells remaining on the upper surface of the membrane were gently scraped off using a cotton swab. The cells that had successfully traversed to the lower surface were fixed with 4% paraformaldehyde for 15 min, stained with 0.1% crystal violet for 20 min at room temperature, and subsequently washed with PBS. Images were captured using an inverted light microscope, and the migratory or invasive capacities were quantified by counting the stained cells in at least three randomly selected fields per insert.

### Quantification of intracellular iron, MDA, and lipid ROS

#### Intracellular iron

To assess the labile iron pool, live cells were loaded with 1 μM FerroOrange probe (DOJINDO) in HBSS for 30 min at 37 °C protected from light. Red fluorescence intensity, reflecting intracellular Fe^2+^ levels, was immediately captured via confocal laser scanning microscopy and quantified.

#### Malondialdehyde (MDA) assay

Intracellular MDA levels, serving as a marker for lipid peroxidation, were evaluated post-treatment (including Acrolein and Erastin exposure) using a specific MDA assay kit (Beyotime, #S0131S) based on the thiobarbituric acid (TBA) method. Absorbance was measured colorimetrically to quantify relative MDA concentrations.

#### Lipid ROS assessment

Cells treated with ferroptosis inducers (Erastin/RSL3) were stained with the lipid peroxidation sensor BDP 581/591 C11 (C11-BODIPY, DOJINDO) according to the manufacturer's instructions. The shift in fluorescence indicating lipid peroxidation was analyzed quantitatively using flow cytometry.

### RIP-qPCR, MeRIP-qPCR, and dot blot assays

#### RIP-qPCR assay

RIP assays were conducted using the PureBinding RNA Immunoprecipitation Kit (GENESEED, P0101). Cell lysates were incubated with 4 μg of anti-TRDMT1 antibody or control IgG conjugated to magnetic beads at 4 °C overnight. Co-precipitated RNA was purified and analyzed by RT-qPCR to assess the interaction between TRDMT1 protein and *TFRC* mRNA.

#### MeRIP-qPCR assay

Total fragmented RNA was subjected to immunoprecipitation using a highly specific anti-m5C antibody (3 μg per reaction; 68301-1-Ig, Proteintech) preconjugated to Dynabeads Protein A/G (Invitrogen). The enriched m5C-modified RNA transcripts were reverse-transcribed, and the relative m5C enrichment on the *TFRC* mRNA was quantified by RT-qPCR.

#### RNA dot blot assay

To detect global m5C levels, serially diluted extracted total RNA (*e*.*g*., 400 ng, 200 ng, 100 ng) was spotted onto a positively charged nylon membrane. The membrane was cross-linked, blocked, and incubated with the anti-m5C antibody, followed by HRP-conjugated secondary antibody incubation and ECL visualization. Methylene blue (MB) staining served as the loading control.

### Dual-luciferase reporter assay

To assess whether TRDMT1 regulates *TFRC* via its 3′ UTR via its 3′UTR, a dual-luciferase reporter assay was performed. The wild-type (WT) or m5C-site mutant (MUT) 3′UTR sequences of *TFRC* were cloned into the pFluc luciferase reporter vector (MiaolingBio). Cells cultured in 24-well plates were co-transfected with the corresponding reporter plasmids and TRDMT1 knockdown constructs using Lipofectamine 3000. After 48 h, luciferase activity was measured using the Dual-Luciferase Reporter Assay System (Promega). Firefly luciferase activity was normalized to Renilla luciferase activity for each sample.

### In vivo tumor xenograft models

All animal procedures were evaluated and approved by the Zhengzhou University Animal Experiment Ethics Committee. Female BALB/c nude mice (4–5 weeks old, GemPharmatech) were randomly divided into specific groups (*e*.*g*., Lv-NC, Lv-TRDMT1, Lv-TRDMT1 + sh-TFRC, or Lv-TFRC; *n* = 6 per group). Mice were subcutaneously injected into the right flank with 100 μl of PBS suspension containing 5 × 10^6^ stably transfected A549 cells. Tumor dimensions were measured every 3 days using a digital caliper, and tumor volume was calculated using the formula: V = 0.5 × Length × Width^2^. After 21 days, mice were euthanized, and the xenograft tumors were excised, photographed, and weighed. Tumor tissues were subsequently fixed in 4% paraformaldehyde for HE staining and IHC analysis (Ki-67, TFRC) to evaluate *in vivo* pathology and target expression.

### Statistical analysis

All quantitative in vitro experiments were independently repeated at least three times. For in vivo experiments, six mice per group were used. Data are presented as the mean ± standard deviation (SD) with individual data points displayed on the bar plots to indicate biological replicates, ensuring data transparency. Statistical analyses were performed using R software (version 4.2) and GraphPad Prism software (version 10.1.2).

For clinical bioinformatics data (*e*.*g*., TCGA-LUAD paired/unpaired tissue comparisons), paired Student's t-tests were utilized for matched tumor-adjacent normal tissues, while unpaired Student's t-tests or Wilcoxon rank-sum tests were applied for non-matched comparisons. For standard experimental differences between two independent groups, a two-tailed, unpaired Student's *t* test was employed. For comparisons among multiple groups, a one-way analysis of variance (ANOVA) followed by Tukey's *post hoc* test was applied. For data involving two independent variables, a two-way ANOVA followed by Sidak's multiple comparisons test was performed. Survival curves were generated using the Kaplan-Meier method, and statistical significance was evaluated *via* the log-rank test. A *p*-value < 0.05 was considered statistically significant (∗*p* < 0.05, ∗∗*p* < 0.01, ∗∗∗*p* < 0.001, ns = not significant).

## Ethics approval and consent to participate

The authors sought and secured approval for this study from the Ethics Committee of the First Affiliated Hospital of Zhengzhou University, adhering to the ethical standards of the Declaration of Helsinki. We obtained written informed consent from all individuals who took part in the research.

## Consent for publication

We have obtained consent to publish this paper from all the participants of this study.

## Data availability

Not applicable.

## Supporting information

This article contains supporting information.

## Conflict of interest

The authors declare that they have no conflicts of interest with the contents of this article.

## Figures and Tables

**Fig. S1:**
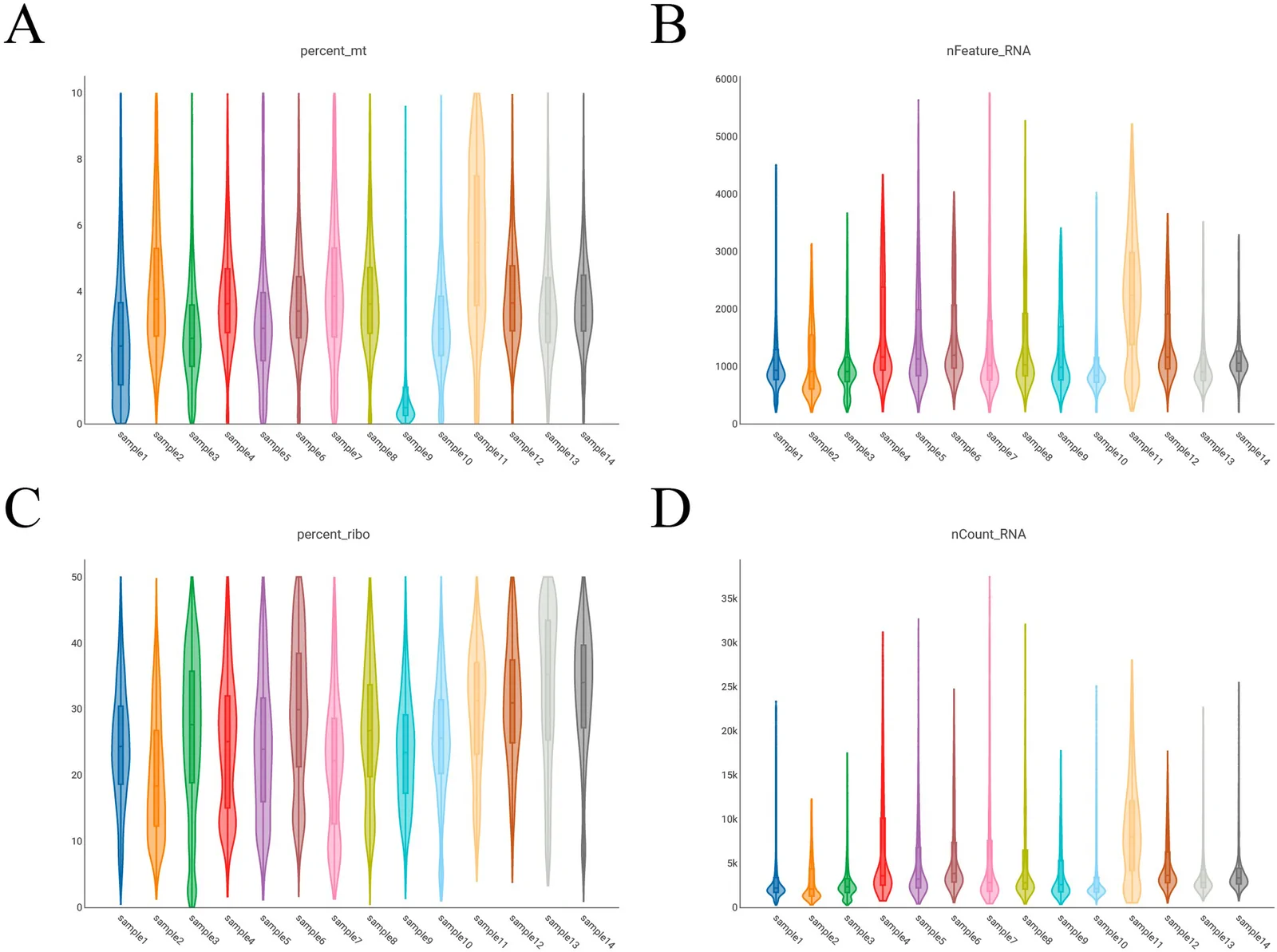


**Fig. S2:**
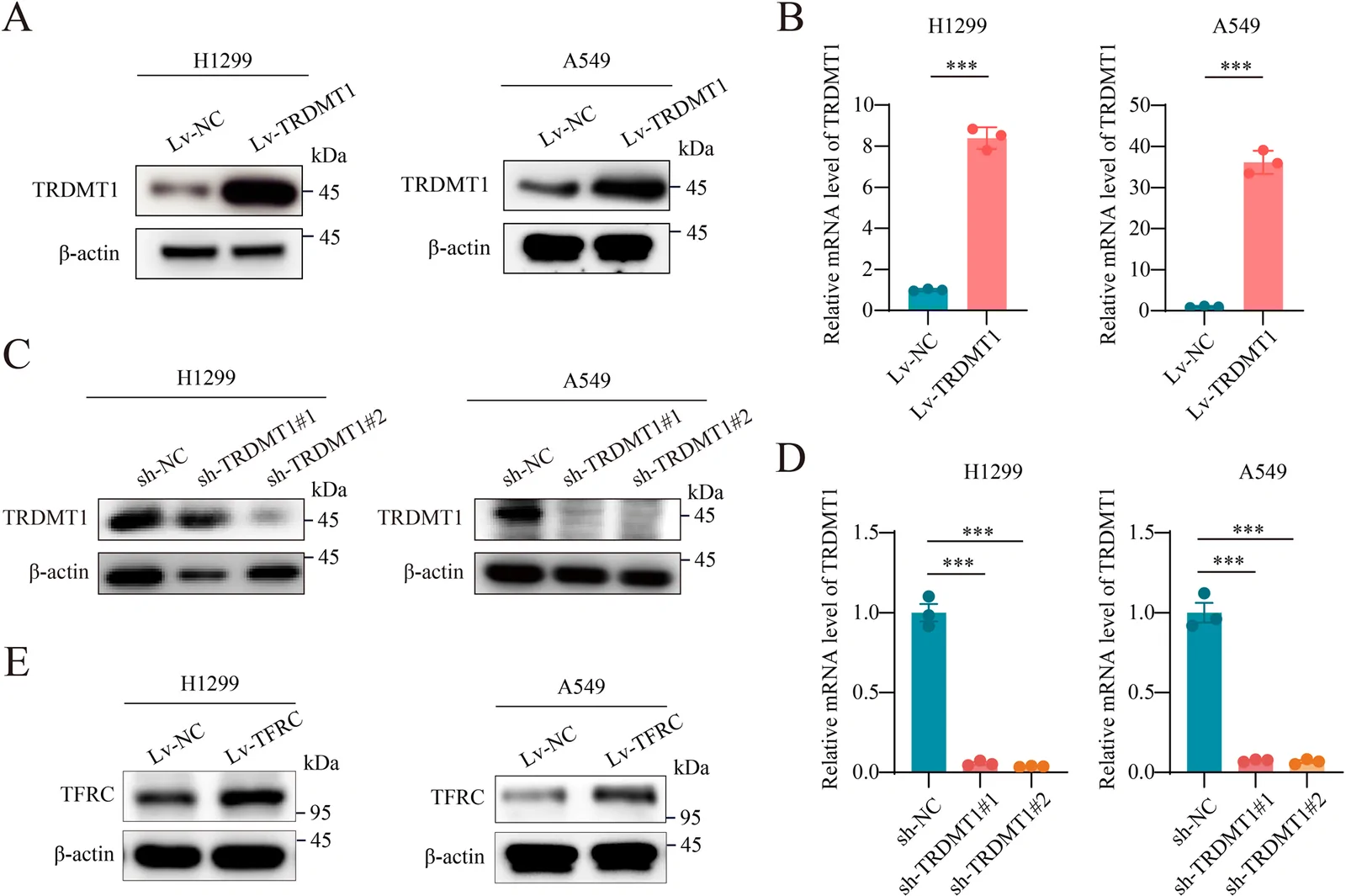


**Fig. S3:**
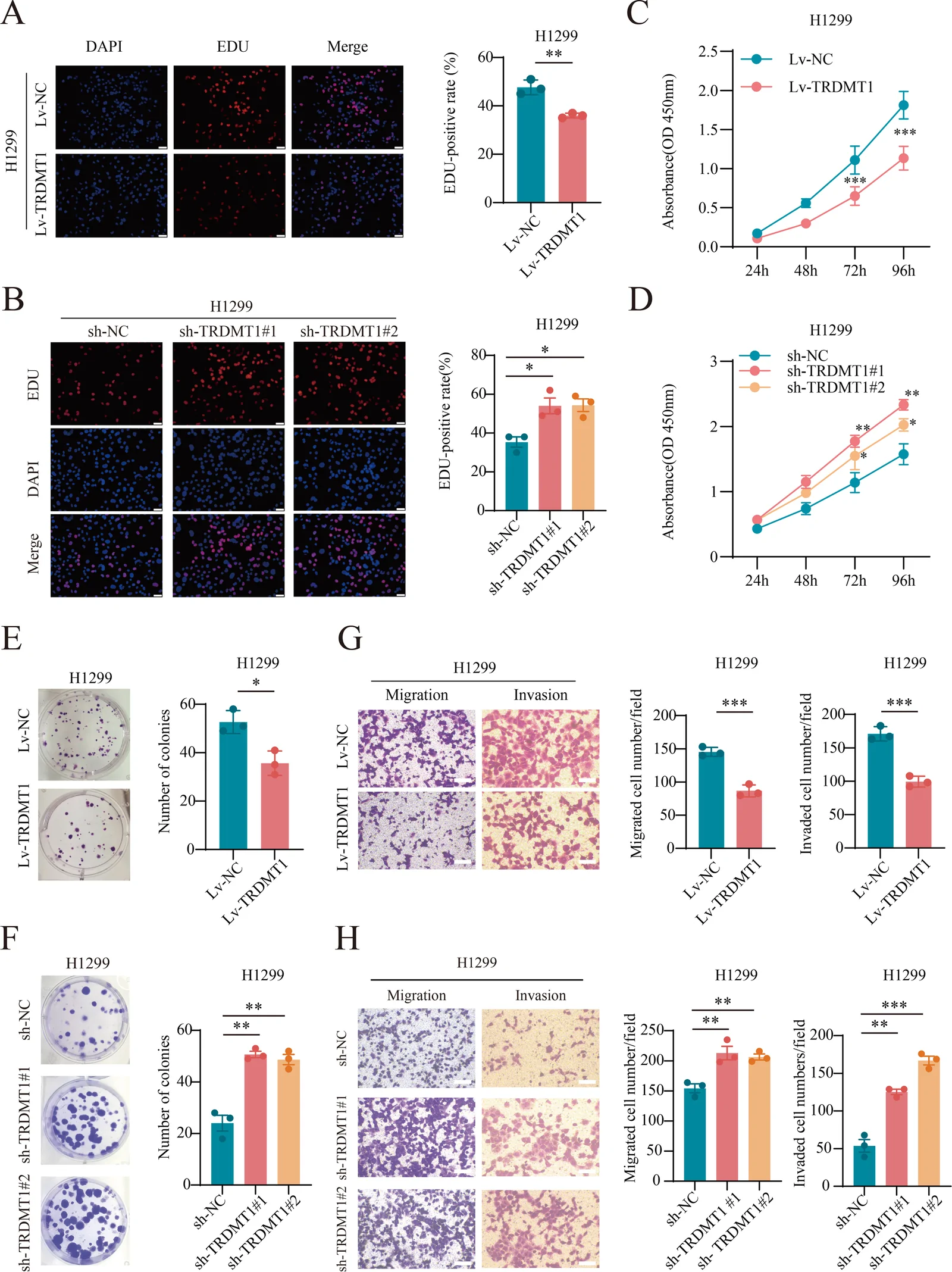


**Fig. S4:**