# Supplementary material for: TRDMT1 slows lung cancer growth by stabilizing TFRC mRNA and triggering ferroptosis cell death
Source: J Biol Chem. 2026 Aug 24;302(10):113474. doi: 10.1016/j.jbc.2026.113474 (PMC13635263; doi:10.1016/j.jbc.2026.113474)
Supplement: Supplementary file 1 [file mmc1.docx]

**Figure S1. Quality control assessment of single-cell RNA sequencing data.**

**(A–D)** Violin plots showing the distribution of key quality control metrics per cell across 14 samples, including the percentage of mitochondrial genes (percent_mt) (A), the number of detected genes (nFeature_RNA) (B), the percentage of ribosomal genes (percent_ribo) (C), and the total UMI (Unique Molecular Identifier) counts (nCount_RNA) (D).

**Figure S2. Validation of TRDMT1 and TFRC overexpression and knockdown efficiencies in LUAD cells.**

**(A, B)** Western blot analysis (A) and relative mRNA expression measured by RT-qPCR (B) of TRDMT1 in H1299 and A549 cells stably transfected with a *TRDMT1* overexpression lentivirus (Lv-TRDMT1) or negative control (Lv-NC). **(C, D)** Western blot analysis (C) and relative mRNA expression measured by RT-qPCR (D) of TRDMT1 in H1299 and A549 cells stably transfected with *TRDMT1* short hairpin RNAs (sh-TRDMT1#1, sh-TRDMT1#2) or negative control (sh-NC). **(E)** Western blot analysis validating the protein overexpression efficiency of TFRC (Lv-TFRC) compared to the empty vector control (Lv-NC) in H1299 and A549 cells. β-actin served as an internal loading control for all Western blot assays. Data are presented as mean ± SD from three independent experiments (n = 3). **P* < 0.05, ***P* < 0.01,*** P < 0.001, determined by two-tailed Student's t-test (B) or one-way ANOVA (D).

**Figure S3. TRDMT1 suppresses oncogenic phenotypes in H1299 cells *in vitro*.**

**(A, B)** EdU assays evaluating the proliferation of H1299 cells following *TRDMT1* overexpression (Lv-TRDMT1) (A) or knockdown (sh-TRDMT1#1 and #2) (B). Representative fluorescence images (left) and quantification of the EdU-positive cell rate (right) are shown. Scale bar = 100 μm. **(C, D)** CCK-8 assays assessing the cell viability of H1299 cells upon *TRDMT1* overexpression (C) or knockdown (D) over a 96-hour period. **(E, F)** Colony formation assays illustrating the impact of *TRDMT1* overexpression (E) and knockdown (F) on the clonogenic ability of H1299 cells. Representative well images and quantitative bar graphs are provided. **(G, H)** Transwell migration (uncoated) and invasion (Matrigel-coated) assays evaluating the metastatic potential of H1299 cells subjected to *TRDMT1* overexpression (G) or knockdown (H). Representative images (left) and quantification of migrated/invaded cells per field (right) are shown. Scale bar = 100 μm. Data are presented as mean ± SD from three independent experiments (*n* = 3). **P* < 0.05, ***P* < 0.01, ****P* < 0.001, determined by two-tailed Student's t-test (A, C, E, G) or one-way/two-way ANOVA followed by appropriate post hoc tests (B, D, F, H).

**Figure S4. Supplementary data supporting the specific regulation of ferroptosis and *TFRC* mRNA stability by TRDMT1.**

**(A–C)** Western blot analysis evaluating the protein levels of key markers for necroptosis (pMLKL, tMLKL) **(A)**, pyroptosis (GSDME) **(B)**, and apoptosis (CASP3) **(C)** in A549 cells following *TRDMT1* knockdown. **(D)** Relative cell viability of *TRDMT1*-overexpressing A549 cells treated with DMSO, Erastin, or a combination of Erastin and Ferrostatin-1 (Fer-1), evaluated by CCK-8 assay. **(E, F)** Relative cell viability **(E)** and MDA levels **(F)** in *TRDMT1*-knockdown A549 cells treated with DMSO or Acrolein. **(G)** Relative pre-mRNA levels of *TFRC* in H1299 cells following *TRDMT1* overexpression, assessed by RT-qPCR. **(H)** Dot blot assays evaluating global m5C RNA methylation levels in A549 and H1299 cells following TRDMT1 overexpression. Methylene blue (MB) staining served as a loading control. **(I, J)** RNA immunoprecipitation (RIP) assay in H1299 cells. Western blot analysis of TRDMT1 protein in the Input, IgG, and IP samples (I), and RT-qPCR quantification of *TFRC* mRNA enrichment in the TRDMT1 immunoprecipitates relative to the IgG control (J). **(K)** mRNA stability assay. H1299 cells (sh-NC and sh-TRDMT1) were treated with Actinomycin D (ActD) to arrest transcription, and *TFRC* mRNA decay rates were measured by RT-qPCR at 0, 4, and 8 hours. **(L, M)** MeRIP-qPCR assay evaluating m5C enrichment on *TFRC* mRNA in H1299 cells following TRDMT1 overexpression **(L)** or knockdown **(M)**. Data are presented as mean ± SD from three independent experiments (*n* = 3). ns, not significant, **P* < 0.05, ***P* < 0.01, ****P* < 0.001, determined by two-tailed Student's t-test (G, J, L), one-way ANOVA (M), or two-way ANOVA (D, E, F, K).
